# Biochemical characterization of the *Helicobacter pylori* bactofilin-homolog HP1542

**DOI:** 10.1371/journal.pone.0218474

**Published:** 2019-06-24

**Authors:** Sven Holtrup, Thomas Heimerl, Uwe Linne, Florian Altegoer, Frank Noll, Barbara Waidner

**Affiliations:** 1 LOEWE Center for Synthetic Microbiology, Philipps-Universität, Marburg, Germany; 2 Faculty of Chemistry, Philipps-Universität, Marburg, Germany; 3 Faculty of Biology, Philipps-Universität, Marburg, Germany; Rijksuniversiteit Groningen, NETHERLANDS

## Abstract

The human pathogen *Helicobacter pylori* is known for its colonization of the upper digestive system, where it escapes the harsh acidic environment by hiding in the mucus layer. One factor promoting this colonization is the helical cell shape of *H*. *pylori*. Among shape determining proteins are cytoskeletal elements like the recently discovered bactofilins. Bactofilins constitute a widespread family of polymer-forming bacterial proteins whose biology is still poorly investigated. Here we describe the first biochemical analysis of the bactofilin HP1542 of *H*. *pylori* reference strain 26695. Purified HP1542 forms sheet-like 2D crystalline assemblies, which clearly depend on a natively structured C-terminus. Polymerization properties and protein stability were investigated. Additionally, we also could demarcate HP1542 from amyloid proteins that share similarities with the bactofilin DUF domain. By using zonal centrifugation of total *H*. *pylori* cell lysates and immunfluorescence analysis we revealed peripheral membrane association of HP1542 mostly pronounced near mid-cell. Interestingly our results indicate that *H*. *pylori* bactofilin does not contribute to cell wall stability. This study might act as a starting point for biophysical studies of the *H*. *pylori* bactofilin biology as well as for the investigation of bactofilin cell physiology in this organism. Importantly, this study is the first biochemical analysis of a bactofilin in a human pathogen.

## Introduction

In recent decades, bacterial cell biology has seen great advances including the discovery of novel cytoskeleton proteins exclusively found in bacteria [1]. A recent addition to these bacteria-specific cytoskeletal proteins are the so-called bactofilins [2]. These small proteins are widespread among most bacterial lineages and involved in a variety of different cellular processes. They are defined by the presence of a central conserved domain of unknown function (DUF583) [3] and flanking N- and C-terminal regions of variable length and sequence, which are often highly charged. A further characteristic of bactofilins is their ability to polymerize spontaneously in the absence of nucleotides or other cofactors [2]. Therefore, these proteins have been proposed to assemble at distinct subcellular sites, serving as multimeric scaffold for the assembly of other proteins. It is supposed that the majority of bactofilin homologues are soluble, except for some enterobacterial orthologs [4] that contain an N-terminal transmembrane region, with the DUF583 domain being located in the bacterial cytoplasm. However due to the spontaneous formation of polymers recombinant protein production often leads to an insolubility of bactofilin polymers, which makes them difficult substrates for crystallographic studies as well as conventional liquid-state nuclear magnetic resonance (NMR) methods. Therefore, considerable attention has been given to this class of cytoskeletal proteins as target for a combination of solid-state NMR and electron microscopy [5]. This technique revealed that the bactofilin BacA of *Caulobacter crescentus* adopt a β-helical architecture, which has not been observed before for any other cytoskeletal filaments [5–8]. Additionally the DUF583 domain of the same bactofilin was structurally analysed by combining a molecular force field with experimental NMR backbone chemical shifts and amino acid contacts obtained by sequence variation [8]. Interestingly, the results of the studies differ in the mode of orientation of the rigid β-helical core, and it is therefore still unclear whether the protein adopt a left-handed or a right-handed orientation [5, 8]. In addition, although this structure has not been reported in other bacterial cytoskeletal filaments, it is similar to that of the fungal prion protein HET-s, which belongs to the class of amyloid proteins [9, 10]. Despite this recent gain of information, the precise cellular function of the bactofilin structures in most organisms is still unknown. Furthermore, the description of the cellular functions reported so far are quite diverse. The first discovered member of this family, the protein CcmA of *Proteus mirabilis*, has been implicated in cell shape and swarming motility [11]. In *C*. *crescentus* and in *Myxococcus xanthus*, it is supposed that bactofilins recruit other proteins to specific subcellular sites. As such the two bactofilin paralogs BacA and BacB of *C*. *crescentus* assemble into membrane-associated polymeric sheets, specifically localized to the cell pole carrying the stalk—a thin protrusion of the cell body involved in cell attachment and nutrient acquisition [2]. As both proteins co-localize with PbpC (Penicillin-binding protein C), a peptidoglycan synthetase, a role in the stalk biogenesis was suggested [2]. In *M*. *xanthus* one of its bactofilin homologs, BacP, assembles into short filamentous structures that emanate from the cell poles, recruiting and thus controlling a small GTPase involved in type IV pili-dependent motility [12]. Whereas the two bactofilins found in *C*. *crescentus* co-polymerize and seem to share a common function, many species produce multiple paralogs displaying clearly distinct roles. Thus, for instance, the four bactofilin paralogues of *M*. *xanthus*, (BacMNOP) harbour distinct functions. Upon the deletion of genes encoding for BacN, BacO, or BacP, cell morphology was found to be normal, while the lack of BacM was shown to be critical for proper cell shape [1]. Recently, a study demonstrated that BacNOP restrain the ParABS chromosome segregation machinery to the subpolar regions of the cell and are therefore involved in proper nucleoid morphology and chromosome segregation [13]. In *Shewanella oneidensis* the protein fusion of SO1662 with mCherry, which is the only bactofilin identified in this organism, assembled as a fluorescent band at midcell suggesting a possible involvement in cell division [2]. In *Bacillus subtilis* it was shown, that the two bactofilins, BacE and BacF, assemble into defined size assemblies that show a dynamic localization pattern and play a role in flagellar assembly [14].

Very recently in *Leptospires* five bactofilin homologs have been identified of which one contributes to the periodic spacing of the cell helix, the integrity of the cell wall, and to motility [15]. Thus, although bactofilins may be versatile scaffolds recruiting and localizing proteins to specific cellular locations, the subcellular positioning and function of bactofilins appears to vary significantly depending on the host organism.

*Helicobacter pylori* is a Gram negative, highly motile, microaerophilic, spiral-shaped organism, which belongs to the class of the epsilon proteobacteria. The natural habitat of this pathogen is the human gastric mucosa and infection of humans results in persistent gastritis, which can develop into peptic ulcer disease and adenocarcinoma [16, 17] [18]. Thereby many factors determine the type and severity of diseases e.g. the status of the host’s immune system, the pathogenicity of *H*. *pylori* strains, and the presence of environmental factors (diet, stress, hygiene level, or the presence of co-infections) [19]. Within these, the helical cell shape is an important feature required for cells to efficiently reach and colonize the gastric mucus [20] [21]. Mutants displaying a rod shaped phenotype were attenuated in stomach colonization [22] and motiliy [23]. In *H*. *pylori*, cell shape maintenance is apparently controlled by at least two unrelated mechanisms that operate at two levels: peptidases influence cell shape by causing peptidoglycan relaxation [22] [24] and four so called *coiled-coil-rich proteins* (Ccrp) depicting cytoskeleton elements influence cell shape most probably by composing an intracellular scaffold [23, 25]. In contrast to the situation in many other bacteria cell morphology in *H*. *pylori* is not influenced by the actin-like protein MreB [25]. In addition one bactofilin homologue was identified, which was found to be important for cell shape maintenance of the characteristic helical cell shape [22]. Infection of mice demonstrated that the *H*. *pylori* bactofilin deficient mutant was strongly outcompeted by wild-type bacteria, identifying the *H*. *pylori* bactofilin as an important pathogenicity factor. However like all bactofilins studied so far the H. pylori bactofilin appears not to be essential for survival of the bacteria itself, indicating that the cells either possess other proteins with redundant functions, or that these proteins are involved in non-essential processes, or both.

However, whether the single *H*. *pylori* bactofilin indeed exhibits biochemical properties of bactofilins and forms a cytoskeleton in this bacterium and, if so, how this putative cytoskeleton is involved in helical cell shape was still unclear. We thus sought to characterize the biochemical properties of the single bactofilin HP1542 *in vitro* and investigate its localization *in vivo*. As such our results provide the first insights into assembly properties and localization of HP1542 and may thus act as a starting point for the investigation of bactofilin cell physiology in this pathogenic organism as well as for designing targeting molecules.

## Material and methods

### Bacterial strains and growth conditions

Bacterial strains are listed in Table 1. *H*. *pylori* strains were routinely cultivated on Dent blood agar in a microaerobic atmosphere as described earlier [26]. Growth experiments were performed in Brucella broth with 5% fetal calf serum (BBF). All growth experiments were performed in triplicate and were repeated at least three times. *E*. *coli* strains were grown aerobically at 37°C in Luria-Bertani medium. When appropriate, growth media were supplemented with 50 μg/l ampicillin, 20 μg/l kanamycin or 20 μg/l chloramphenicol respectively. Ellipsoid testing was performed according to the manufacturer’s instructions.

**Table 1:** List of strains and primer used in this study.

**Strains: *H*. *pylori***		
**Name**	**Description**	**reference**
26695	wt	[27]
G27	wt	[28]
1061	wt	[29]
26695_1542KM	26695 *hp1542*::*Pneo*	This study
G27_1480KM	G27 *hp1542*::*Pneo*	This study
1061_1542KM	1061 *hp1542*::*Pneo*	This study
**Strains: *E*. *coli***		
**Name**	**Description**	**reference**
DH5α	*E*. *coli* K12 derivate, *fhuA2 lac(del)U169 phoA glnV44 Φ80' lacZ(del)M15 gyrA96 recA1 relA1 endA1 thi-1 hsdR17*. Used for molecular cloning	Bethesda Research Laboratories
BL21(DE3)	*E*. *coli* BL21 derivate, *dcm ompT hsdS(rB-mB-) gal* carrying the λDE3 prophage. Used as host for heterologous expression of *H*. *pylori* genes	Stratagene
**Primer**		
**Name**	**Sequence**	**reference**
HPG27_1480-80u-5	CAG CCC ATA AAC CCC ATG AGT	[22]
NEO-1542-L1	CCT AGA TTT AGA TGT CTG GAA ACT CGC CCT AAG AAT A	This study
1542-PNEO-R1	CGT ACC GGT TCC AAT TTT GCC ATC TAC ATG CAA ATG GT	This study
HP1542up BsaI	ATG GTA GGT CTC AGC GCA TGG CAA TCT TTG ATA ACA ATA ATA AAT	This study
HP1542dw BsaI	ATG GTA GGT CTC ATA TCA TTT ATT TTC AAT TTT CTT TTC TTG CTC A	This study
Hp1542_F:	AAT TCC ATG GGC GCA ATC TTT GAT AAC	This study
Hp1542-6H_R	AAT TGG ATC CTT AAT GGT GAT GGT GAT GGT GTT TAT TTT CAAT TTT	This study
1542_112_AUG_BsaI_for	ATG GTA GGT CTC AGC GCA TGC ACG TAG ATG GCG AAT TAG	This study
1542_339_BsaI_rev	ATG GTA GGT CTC ATA TCT ATT CCC CAA TCA AAA TCC CC	This study

### DNA techniques and mutagenesis of *H*. *pylori*

Restriction and modifying enzymes (New England Biolabs, USA) were used according to the manufacturer’s instructions. Cloning was performed in *E*. *coli* according to standard protocols. Plasmids were isolated with a QIAprep Spin Miniprep Kit from Qiagen (Qiagen, Hilden). To generate plasmids pASK7_1542, pASK7_1542__1–339_ and 1542__112–411_ the coding sequence of *H*. *pylori* strain 26695 was amplified using the primer pairs listed in Table 1 and cloned via the *Bsa*I restriction sites added as 5'-extensions into plasmid pASKIBA-7 (IBA, Göttingen). To generate plasmid pET24d-Hp1542-6HC the coding sequence of *H*. *pylori* HP1542 was cloned into the pET24d(+) vector (Novagen) at the NcoI/BamHI sites. The C-terminal His_6_-tag fusion was achieved using primer extensions.

The isogenic *H*. *pylori* HP1542 deletion mutant were constructed as described earlier [25, 26]. Briefly, the resistance marker gene (Pneo) was fused to upstream and downstream DNA regions of mutagenized genes by using a modified version of the megaprimer PCR protocol [30, 31] and primers listed in Table 1. Subsequently marker exchange mutagenesis of *H*. *pylori* was performed according to standard procedures [32]. *H*. *pylori* mutants carrying the resistance genes inserted into the chromosome were selected by growth on Dent blood agar containing kanamycin at concentrations of 20 mg/l. The correct insertions were verified by PCR and sequencing.

### Production and analysis of recombinant proteins

Recombinant versions of the *H*. *pylori* HP1542 protein with different tags fused to the N-terminus were produced in *E*. *coli* using either the Streptag protein expression system from IBA (Göttingen, Germany) or the IPTG inducible pET system from Novagen. Both systems were used according to the manufacturer’s instructions (http://www.iba-go.de, http://www.merckmillipore.com). Briefly, the plasmids were transferred to *E*. *coli* BL21 (DE3) (Novagen) and expression was induced with 0.2 mg/l anhydrotetracycline and 1 mM IPTG respectively. After cell lysis by a Microfluidizer (M110-L, Microfluidics), cell debris was removed by high-speed centrifugation. Recombinant proteins were purified to homogeneity on a Strep-Tactin column or by Ni-ion affinity respectively. Further purification and analysis were achieved *via* size-exclusion chromatography using either a Superose 6 10/300GL column (Tricorn), a Superdex 200 10/300 GL, Superdex 200 Increase 10/300 GL or a Superdex 75 Increase 10/300 GL/GE (GE Healthcare) with buffers listed in Table 2. Standard buffer for purification were buffer W (IBA) for Strep-1542 and SEC buffer respectivly. Protein size was estimated using Bio-Rad’s gel filtration standard containing thyroglobulin (670 kDa), γ-globulin (158 kDa), ovalbumin (44 kDa), myoglobin (17 kDa) and vitamin B12 (1.35 kDa).

**Table 2:** List of plasmids used in this study.

Plasmids		
Name	Relevant characteristics	Reference
pASK7_1542	pASK-iba7 expression vector carrying *hp1542* ORF under control of the Tet-promotor, inserted in *Bsa*I restriction site	This study
pET24d-Hp1542-6HC	Pet-24d expression vector carrying *hp1542* ORF, fused to the sequence of a 6-His tag, under control of the T7-promotor, inserted between *Nco*I and *Xho*I restriction sites	This study
pZErO-2	Cloning vector, MCS in *lacZ*_ *neo*, Kmr	Invitrogen
pZErO-2_hp1480	pZEro-2 containing the Sequence of *hp1480* for *hp1480* (*hp1542)* deletions by vector integration through single cross-over	This study
pASK7_1542__1–339_	pASK-iba7 expression vector carrying bp 1 to 339 of hp1542 ORF under control of the Tet-promotor, inserted in BsaI restriction site	This study
pASK7_1542__112–411_	pASK-iba7 expression vector carrying bp 112 to 411 of hp1542 ORF under control of the Tet-promotor, inserted in BsaI restriction site	This study

#### Atomic force microscopy

AFM images were taken in tapping mode in liquid at room temperature with a MultiMode VIII AFM equipped with a Nanoscope 8.0 controller (Bruker Nano Surfaces Division) using SNL cantilevers (k = 0.12 N/m, f_0_ = 23 kHz, Bruker). Protein samples in different imaging buffers (300 mM KCl, 10 mM Tris-HCl, varying pH) were allowed to adsorbe onto freshly cleaved mica or HOPG surfaces as substrates, respectively. Prior to incubation the chosen substrate was glued onto a round metal disk which was magnetically fixed onto the AFM scanner (PicoForce scanner, Bruker). Before the MultiMode measuring head equipped with a liquid cell (Bruker) and the cantilever was put onto the stage, additional imaging buffer was applied to the liquid cell between cantilever and glass body to prevent formation of gas bubbles close to the cantilever. Afterwards, the whole systems was allowed to reach thermal equilibrium (30 mins.) before taking images. Image processing was performed with NanoScope Analysis V1.90R1 software package.

#### Cell fractionation studies of cell lysates

Cell fractionation studies were performed as described earlier [2]. Briefly, membrane vesicles isolated from wild-type 26695 were incubated for 1 h in 100 mM NaCO_3_ and pelleted by ultracentrifugation. Samples from the crude protein extract, the total membrane fraction, the supernatant as well as the pellet obtained after centrifugation were analyzed by immunoblotting using anti-HP1542 and anti-CagT antibodies.

#### Cross-linking assay

For cross-linking HP1542 *in vitro*, 10 μl of glutaraldehyde dilutions ranging from 10% to 0.01% were added to 90 μl of the strep-tagged protein and incubated for 10 min at 23°C. The reaction was stopped by adding 20 μl Tris 0.5 M (pH 7). 20 μl of each sample was directly analyzed by SDS-PAGE and immunoblotting.

#### Circular dichroism (CD)

CD-spectroscopy was performed with a Jasco J-810 spectrophotometer. Samples were prepared in 25 mM Tris buffer with varying pH-values. CD-spectra were measured in the range from 250–190 nm with a scanning speed of 50 nm/min and standard sensitivity of 100 mdeg, using a 1 mm rectangular quartz cell. Data points were subtracted from a background reading of the buffer, and the molar ellipticity was calculated. Data are presented as the molar ellipticity (deg cm^2^/dmol) at each wavelength (nm).

#### ESI-TOF-MS

Size exclusion purified proteins were dialysed against 100 mM ammonium acetate (pH 7.6). Subsequently, samples were infused through the instruments internal sample syringe. Positive ions within the mass range of 500–12.000 m/z were detected. Data were acquired in manual mode without automatic mass drift correction. Averaged spectra were deconvoluted after baseline subtraction and eventually smoothing using MassLynx instrument software with MaxEnt1 extension.

#### Proteinase K digestion

Purified Strep-HP1542 (2 mg/ml) were incubated in present of increasing concentrations of Proteinase K (0.0000001–1 mg/ml; Sigma) for 10 min at 37°C. The reaction was stopped by boiling the samples in SDS-sample buffer at 95°C for 10 min. Subsequently, the samples were directly analyzed by SDS-PAGE and Coomassie staining.

#### Spin down assays

Spin down assays were performed as follows: 40 μl of purified protein fractions in buffer were centrifuged at 13000 rpm in a bench centrifuge and split in two fractions: the upper 20 μl were defined as soluble fraction and the remaining 20 μl were defined as pellet fraction. SDS sample buffer were added and equal volumes of supernatant and pellet were subjected to SDS PAGE analysis.

#### Western blotting

Western Blotting were performed using either Strep-Tactin HRP conjugate (IBA, Göttingen), or anti-Strep (BIO-RAD) and anti-His (Roche) antibodies in combination with a second anti mouse-HRP conjugate (Sigma-Aldrichs) or anti-1542 peptide antibody (Davids Biotechnology, Regensburg) in combination with a second anti rabbit-HRP conjugate (Sigma) respectively. 20 μg of protein verified by Bradford tests were subjected to gel electrophoresis and successively blotted on nitrocellulose membranes. Bound antibodies were detected with enhanced chemiluminescent (ECL) detection reagents as substrates followed by incubation for 2 min and chemoluminescence detection with ChemiDoc MP System (BIO-RAD).

#### Zonal sedimentation analysis

Size exclusion purified proteins were applied to a 9 ml sucrose gradient (5–15%) in buffer W (100 mM Tris (pH 8), 150 mM NaCl). Centrifugation was performed using the Beckman Coulter Optima XPN-80 ultracentrifuge with a SW40Ti swing rotor and centrifuged for 16 h at 38.000 x g and 4°C. The gradients were fractionated from the top into 0.5 ml aliquots. Samples from each fraction were analyzed by 12% SDS-PAGE. For calibration curves, the protein standard (BioRad) containing a mixture of thyroglobulin (670 kDa, 19S), bovine gamma-globulin (158 kDa, 7.4 S), chicken ovalbumin (44 kDa, 3.5 S), equine myoglobin (17 kDa, 2.0 S), vitamin B_12_ (1.35 kDa) were used. Native weight was calculated according to the equation M = 3.909S x Rs (Stokes radius, nm).

### Electron microscopy

Carbon coated copper grids (400 mesh) were hydrophilized by glow discharging (PELCO easiGlow, Ted Pella, USA). 5 μl of a protein suspension with a concentration of 15 μg/ml was applied onto the hydrophilized grids and stained with 2% uranyl acetate after a short washing step with H_2_Obidest. Samples were analyzed with a JEOL JEM-2100 transmission electron microscope using an acceleration voltage of either 80 or 200 kV. For image acquisition a F214 FastScan CCD camera (TVIPS, Gauting) was used.

### Immunofluorescence

Immunofluorescence of *H*. *pylori* cells was performed as described earlier [23, 26, 33] with the following modifications: anti-1542 antibody (different dilutions) was used as primary antibody, which was detected by the secondary antibody goat anti-rabbit Alexa Fluor 488 (Invitrogen) (1:100). Fluorescence microscopy was performed on a Zeiss Axioobserver Z1 microscope using a 100× objective with a numerical aperture of 1.45. Images were acquired with a digital Cascade electron microscopy charge-coupled-device (EM-CCD) camera (Photometrix). Image analysis was performed using BacStalk [34].

### Testing of cell wall integrity

Ampicillin and Amoxicillin sensitivity was determined by plating 200 μL of overnight culture on agar plates lacking antimicrobials and applying E-test strips (AB Biodisk). Plates were incubated for 2–3 days and read according to the manufacturer’s instructions. Lysozyme killing assays were peformed as described in Wang *et al*. [35]. Briefly, *H*. *pylori cells* grown on agar plates to late log phase were suspended in PBS (phosphate-buffered saline) to a concentration of ~10^9^ cells/ml. Upon addition of lysozyme (final concentration of 30 mg/ml), the cell suspensions were incubated at 37°C under 2%O_2_ with shaking. Samples were then removed at various time points (0, 2, 4, and 6 h), serially diluted, and spread onto DENT agar plates. Colony counts were recorded after 4 days of incubation in a microaerobic atmosphere at 37°C. Hyperosmolarity experiments were performed by comparing the growth of the wild type versus HP1542 deletion mutant in triplicate cultures with and without addition of sodium chloride in two independent experiments as described elsewhere [36].

## Results

### The bactofilin HP1542 of *H*. *pylori* is a soluble protein, which eluted as monomer from size exclusion chromatography

**Fig 1:**
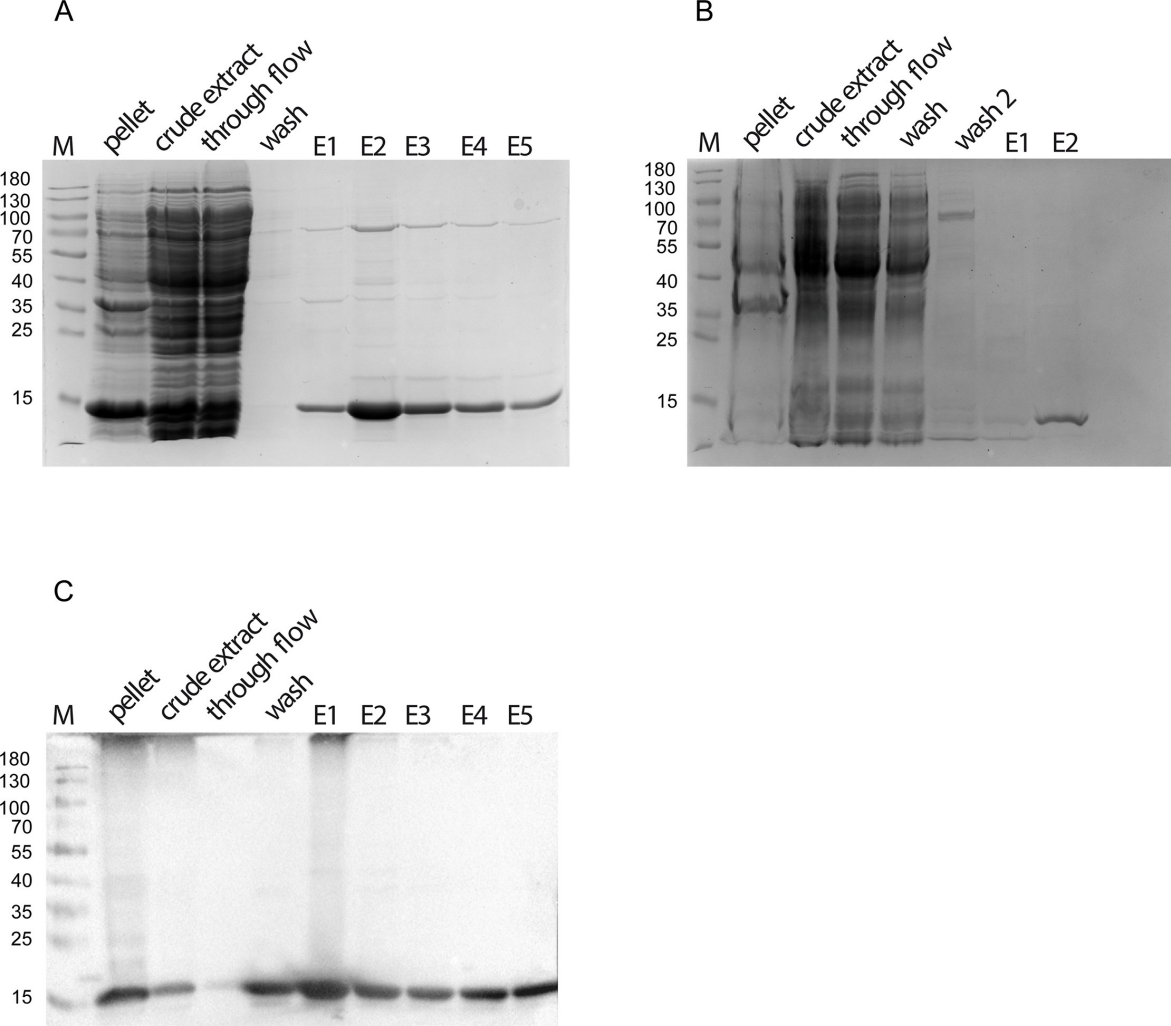
SDS-PAGE analysis of recombinantly produced Strep-HP1542 and HP1542-His from *E*. *coli* BL21. Samples were analyzed on 12% discontinuous polyacrylamide gels. Lane M; low molecular weight marker proteins (Thermo scientific) (A) analysis of recombinant Strep-HP1542 and HP-1542-His (B) through different purification steps. Lanes pellet/ crude extract: cell lysate before purification. Lane through flow: flow through of the cell lysate. Lane wash/ wash 2 after-binding column wash fraction(s). Lane E(n): elution fraction n of elution. (C) Corresponding western blot analysis to (A).

**Fig 2:**
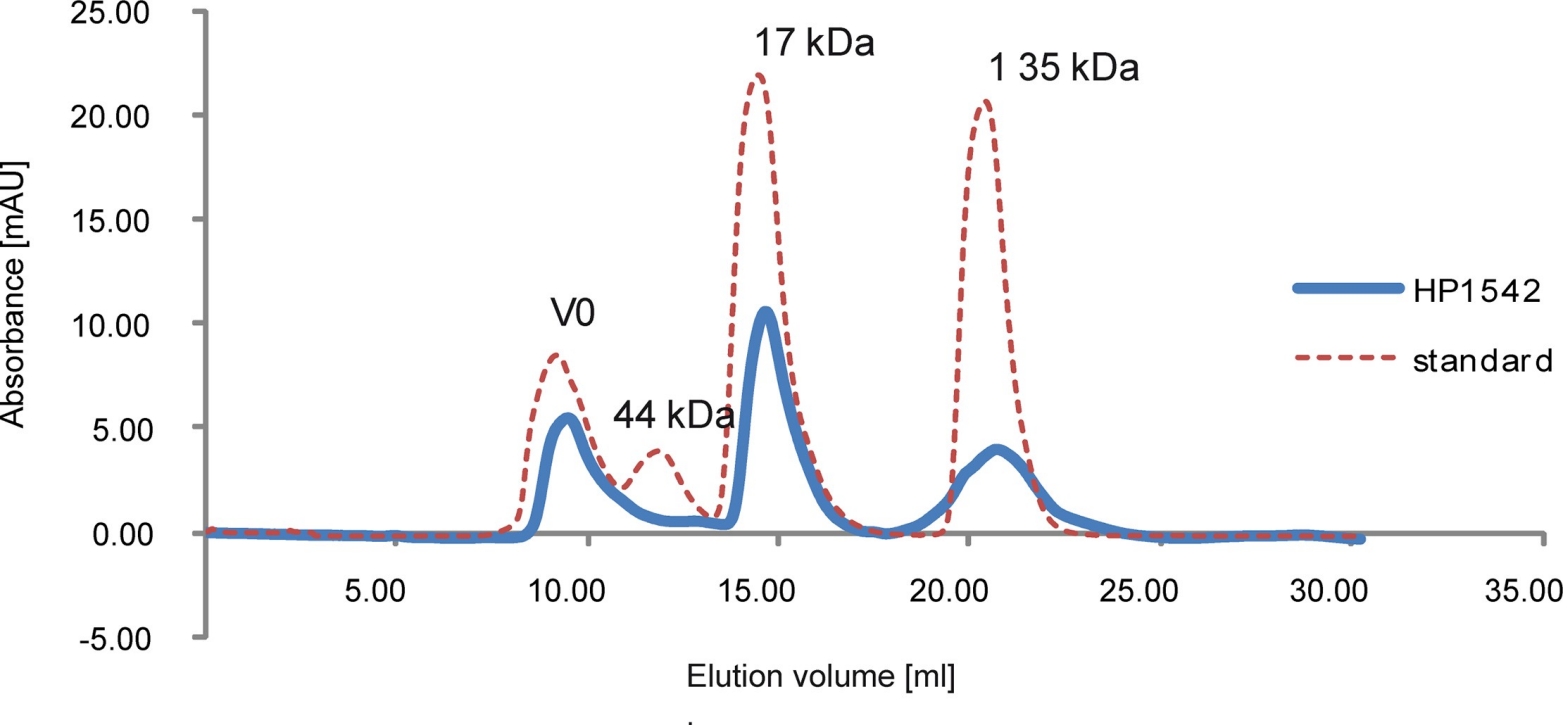
Elution profile on Superdex 75 Increase 10/300 GL/GE of HP1542. Elution profile on Superdex 75 Increase 10/300 GL/GE (GE Healthcare) of affinity-purified Strep-HP1542 (blue line) and standard proteins (red dashed line, Bio-Rad) as follows: thyroglobulin (670 kDa, 19 S), bovine gamma-globulin (158 kDa, 7.4 S), chicken ovalbumin (44 kDa, 3.5 S), equine myoglobin (17 kDa, 2.0 S), vitamin B12(1.35 kDa). Molecular weights are indicated above. mAU, milliabsorbance units at a wavelength of 280 nm.

In order to obtain insights into the biochemical properties of the single bactofilin in *H*. *pylori*, we purified the protein HP1542 of the reference strain 26695 as N- and C- terminal tagged versions. Although it was previously published that the *H*. *pylori* bactofilin forms high-molecular aggregates when overexpressed in *E*. *coli* [37] in our hands HP1542 could be purified as a soluble protein under standard conditions. This was demonstrated on SDS-PAGE by using either a N- terminal Strep-tagged (Fig 1A) or a C- terminal His_6_-tagged version of HP1542 (Fig 1B) and could be verified via Western Blotting (Fig 1C). Further analysis using size exclusion chromatography (SEC) and different columns revealed that most HP1542 eluted in the monomeric form as demonstrated in Fig 2 for the Strep-tagged version by a single peak at about 15 to 17 ml (Fig 2), which corresponds to the size of Myoglobin (17kDa) of the gel filtration standard. This result is in good agreement to the calculated molecular mass of about 16 kDa, including the fusion tags (16.35 kDa for Strep-tagged version and 15.39 kDa for the His_6_-tagged version). In addition HP1542 was also found in the void volume. To confirm the molecular mass of the purified Strep-tagged HP1542 the corresponding SEC sample was analyzed with the help of electrospray-ionization time-of-flight mass spectrometry (ESI-TOF MS) after dialysis against 100 mM ammonium acetate. In a variety of studies it has been demonstrated that noncovalent protein complexes such as protein oligomers and protein‐ligand complexes can be detected by using this method [38]. S1 Fig illustrates the deconvoluted electrospray mass spectrum of Strep-HP1542 demonstrating the mass of 16220 Da with very few additional masses present. As such the mass of 16220 perfectly matches the calculated mass of Strep-HP1542 without the starting methionine (16351.71 Da– 131 Da = 16220 Da). This result shows that the purified Strep-HP1542 was highly pure and remained its monomeric character even after dialysis.

### HP1542 is able to polymerize spontaneously in the absence of nucleotides or other cofactors

**Fig 3:**
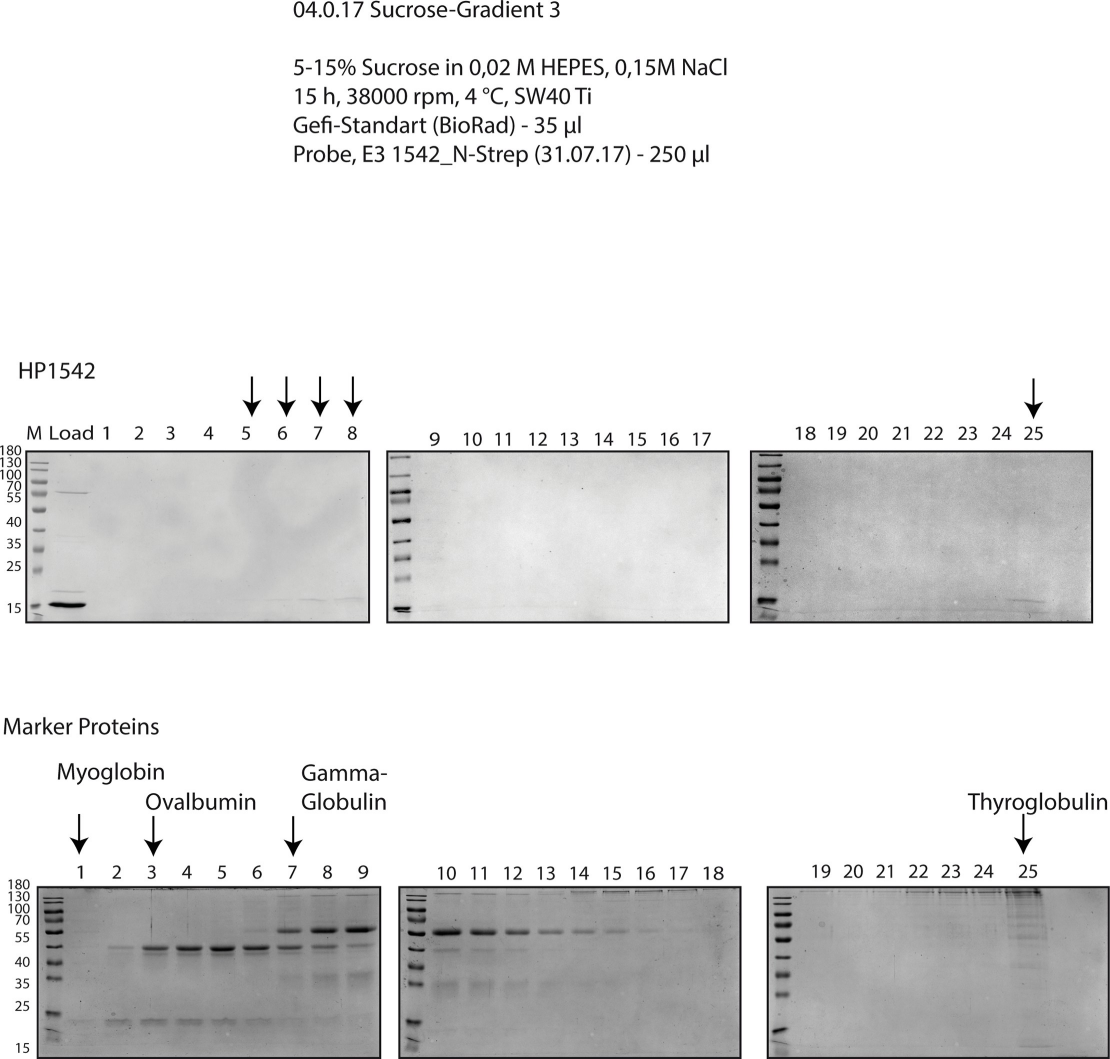
Zonal gradient centrifugation. Zonal centrifugation of Strep-HP1542 (upper panel) and gel filtration standard mix (Bio Rad, lower panel) through 5–15% sucrose gradient. Equal amounts of sample fraction were loaded on SDS-PAGE and stained with Coomassie. Strep-HP1542 containing fractions and standard proteins were indicated by arrowheads and names respectively.

**Fig 4:**
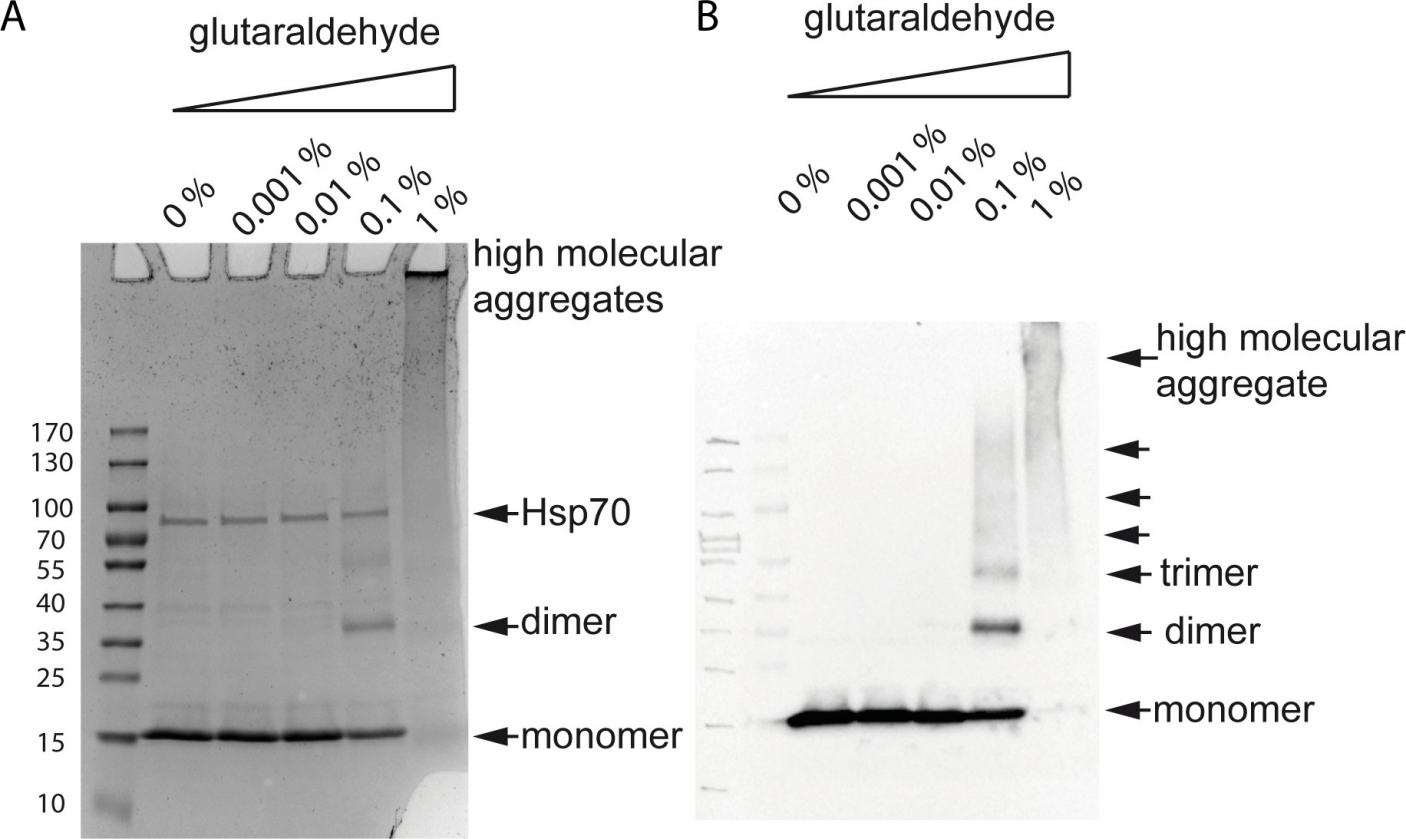
Chemical crosslinking assay. SDS-PAGE (A) and western blot (B) analysis of cross-linked recombinant Strep-HP1542. Respective concentrations of glutaraldehyde are indicated above. Arrowheads depict detected signals of Strep-HP1542 monomers, dimers as well as higher molecular aggregates. The Hsp70 contamination which is seen in the Coomassie stained SDS PAGE is marked.

A characteristic of bactofilins is their ability to polymerize spontaneously in the absence of nucleotides or other cofactors [2]. Therefore, we additionally carried out analytical ultracentrifugation, a powerful tool for the characterization of protein self-association. Accordingly, purified Strep-HP1542 was subjected to sucrose gradient ultracentrifugation, the distribution in the gradient was analyzed by SDS-PAGE and compared with that of marker proteins in the same approach (Figs 3 and 4). Interestingly Strep-HP1542 was most prominently visible in fractions corresponding to proteins of higher molecular mass (Fig 3). No clear signals were found in fractions belonging to Myoglobin (17kDa). HP1542 was also found in the fraction coinciding to the highest molecular weight proteins standard protein thyroglobulin (670 kDa). The same results were obtained using HP1542-His_6_. To further dissect the oligomeric state of the *H*. *pylori* bactofilin we performed protein cross-linking experiments using glutaraldehyde. Exposure to glutaraldehyde in the range of 0.1% resulted in the appearance of clear signals at an apparent molecular mass of 32 kDa and 48 kDa respectively, which could be interpreted as possible cross-linked dimeric and trimeric species (Fig 4A). Additionally a diffuse signal at an apparent molecular mass of more than 170 kDa resembling a higher-ordered oligomeric structure was visible at glutaraldehyde concentrations of 1%. As these cross-linking experiments were performed with elution fraction containing the higher molecular band proved to be the Hsp70 chaperon, we confirmed the existence of HP1542 oligomers via western blotting (Fig 4B). Our results demonstrate that HP1542 is able to undergo stepwise oligomerization indicating that HP1542 like other bactofilins is able to polymerize spontaneously in the absence of nucleotides or other cofactors.

**Fig 5:**
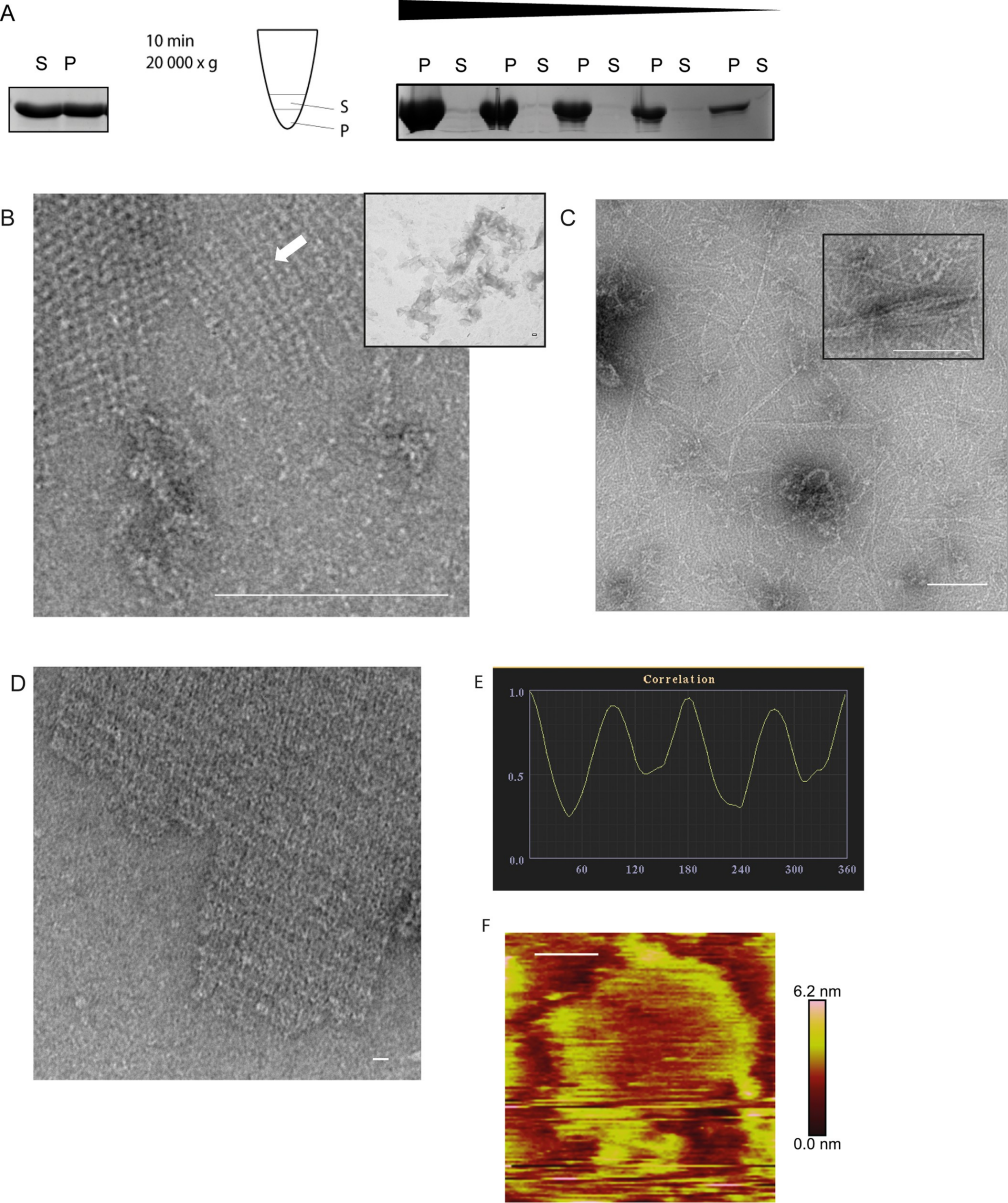
Ultrastructure of HP1542. (A) Spin-down assay of HP1542 after purification from *E*. *coli*. Left: coomassie-stained SDS-PAGE of the spin-down assay of freshly purified Strep-HP1542. Middle: pictogram demonstrating the procedure. Samples were split in two equal fractions: the upper one was defined as soluble fraction (S) and the remaining one was defined as pellet fraction (P). Right: coomassie-stained SDS-PAGE of the spin-down assays with decreasing protein concentrations of aggregated Strep-HP1542; (B-D) Transmission electron microscope (TEM) images of purified Strep-HP1542 in high (B, large image) and low magnification (B, small image), of purified HP1542-His (C) and of purified HP1542 without tag (D). (E) Example of symmetry determination of a unit cells via correlation averaging using the ANIMETRA software. (F) Atomic Force microscopy (AFM) topographic image of HP1542 assembled on mica. A height scale bar is shown to the right. Scale bars of all microscopic images are 100 nm.

**Fig 6:**
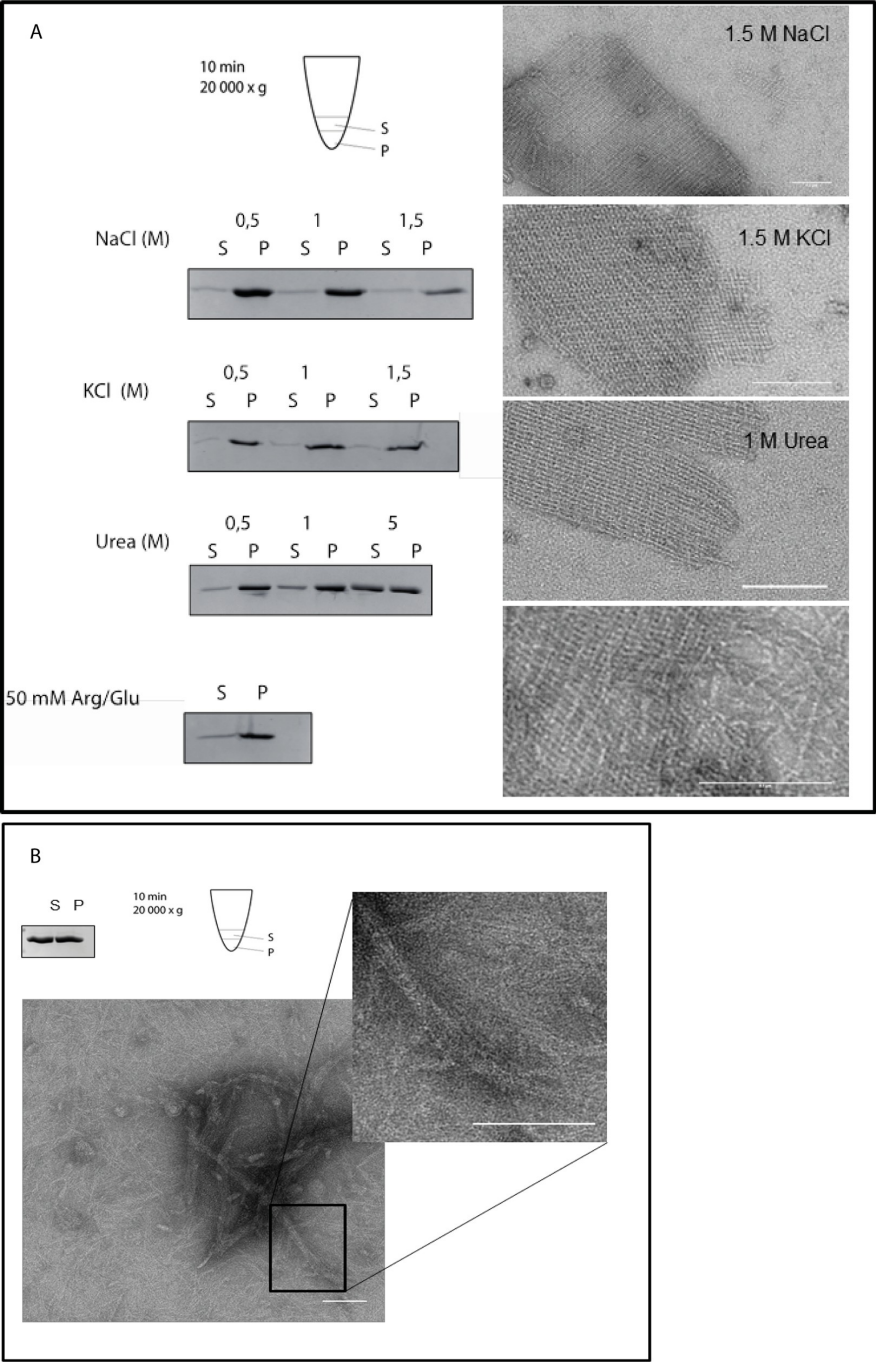
Evaluation of 2-D crystalline protein assembly of HP1542. (A) Evaluation of aggregation and 2-D crystalline protein assembly of recombinant Strep-HP1542 in low spin-down assays (left panels) and with electron microscopy (right panels) after various treatments. Conditions were indicated. (B) Upper panel: Spin-down assay of HP1542_1-339_ after purification from *E*. *coli*. Pictogram is demonstrating the procedure. Samples were split in two equal fractions: the upper one was defined as soluble fraction (S) and the remaining one was defined as gel-like-pellet fraction (P). Lower panel: transmission electron microscope (TEM) images of purified HP1542_1-339_ in low (and higher magnification (inlet)) Scale bars 100 nm.

To get an idea of the time needed for oligomerization purified HP1542 was subjected to low-spin centrifugation (13,000 rpm). As no clear pellet was visible after this kind of centrifugation, we split the sample in two equal fractions: the upper one was taken out and defined as soluble fraction and the remaining one was defined as gel-like pellet fraction (Fig 5). Thus almost same amounts of protein were found in freshly purified samples indicating the existence of complete solubility (Fig 5A). However, the amount of protein shifted towards the gel-like pellet fraction during the next days (4°C, without shaking). No clear reproducible time dependency could be observed indicating more influencing parameters. Likewise no clear correlation between protein concentration and oligomerization could be found (Fig 5A). This behavior was also unchanged in a wide range of different buffer systems (Tab.2 and Fig 6A) indicating robustness and polymerization independence from cations as it was seen for other bactofilins [2, 7]. Solely addition of 1 M urea restored the solubility of HP1542 without denaturing the protein as demonstrated via electron microscopy (Fig 6 and see below).

In addition we subjected the purified HP1542 to electron microscopy. Interestingly, irrespective of sample age and despite eluting as a monomer from SEC, HP1542 formed extended filamentous structures *in vitro*, in the absence of any added cofactor and independent of the buffer system used (Figs 5 & 6). In particular 2D crystalline assemblies were the most abundant higher ordered structures in the sample (Figs 5 & 6). These assemblies demonstrated an amazing regular ribbon-like pattern that seemed to be composed of very regular ordered subunits. This kind of pattern was also observed for BacA form *C*. *crescentus* and described as a woven carpet-like pattern with a distance between two adjacent “knots” of 5.6 nm [5]. Thus we also measured the “knots” distances of the HP1542 woven carpet-like pattern in several images. The measured average distance of about 5 nm confirmed the visual similarity. Notably BacA of *C*. *crescentus* and HP1542 share only a very low sequence similarity of 24% (analyzed via NCBI BLASTP (https://blast.ncbi.nlm.nih.gov/Blast.cgi?PAGE=Proteins)) despite of both having the characteristic bactofilin domain (DUF583) (S2 Fig). By comparing the electron microscopy images of the two tagged protein versions of purified HP1542 we noticed that these 2D assemblies were clearly more observed in solution of Strep-1542 rather HP1542-His (Fig 5B vs 5C). In order to rule out that these structures might be artificial caused by the protein tag, we cleaved the Strep-tag of Strep-HP1542 by using factor Xa. Subsequent electron microscopy images clearly demonstrated that the observed 2D crystalline structures were most prominent without any tag (Fig 5D). Thus this result suggests that the His_6_-tag exerts a negative influence on the assembly properties. With respect to this result, we focused on Strep-HP1542 during further investigations.

A closer inspection of these 2D structures revealed that the 2D sheets most likely are built of protofilaments of about 3 nm in width, which were seen next to the crystalline arrays (Fig 5 6B arrow). Visually, protofilaments show a high propensity to interact laterally, thereby forming these large assemblies. Interestingly, the same diameter was observed for both BacA and BacM [2, 5, 7] depicting similarity of bactofilins. However, it was not possible to define a typical length by measuring the length of these filaments suggesting that there is no favorite oligomeric state of HP1542 before assembling into 2D sheets.

Interestingly, the structure of the HP1542 protein sheets appears amazingly symmetric (Fig 5), which is why we further analyzed the electron microscopy images with the ANIMETRA CRYSTALS software package (release 1.1; ANIMETRA). Correlations averaging of electron micrographs demonstrate that the protein sheets most probably have a simple P2 symmetry (Fig 5E).

To gain further insights in the architecture and the biophysical properties of the HP1542 polymeric sheets at nanometer resolution, we applied the purified Strep-HP1542 protein to atomic force microscopy (AFM). AFM is a versatile tool that allows imaging of the sample surface with nanometer resolution and also to determine the flexibility or elasticity by using force spectroscopy. Unfortunately, although testing different settings it was not possible to achieve nanometer resolution as protein subunits seem to spread upon contact *via* tapping mode with the cantilever. However, larger assemblies could be visualized and demonstrated an average height of about 5 nm of the polymeric protein structure suggesting a single or double protein layer rather than a multilayer (Fig 5F). Furthermore force distance measurements using PeakForce Tapping (PFT, Bruker) revealed that even analysis with a force of as low as 10 pN gave no reliable results as the HP1542 polymers tend to stick to the cantilevers used. However, the analysis of the biophysical properties of these amazing protein sheets by using AFM will be subject of our ongoing studies.

Lateral interactions of BacM filaments were reported to be disrupted by the presence of anionic glycine [7]. However addition of 20 mM glycine did not change the HP1542 2D crystalline structures. Likewise cations were known to influence the assembly of a lot of polymer forming proteins like self-assembling intermediate filaments [39]. Thus we wished to analyze whether there is any ion-specific control of the self-assembly dynamics of the 2D crystalline protein sheets of HP1542. Purified HP1542 was subjected to dialysis to different conditions (listed in Table 2) and further analyzed by both low-spin centrifugation (13,000 rpm) and electron microscopy. In addition different pH values ranging from pH 5 to pH 9.5 were considered taking the calculated pI of pH 6.2 of HP1542 into account. Notably, despite its habitat in the human stomach *H*. *pylori* is a neutralophile bacterium with near neutral pH of the cytoplasm [40]. However neither addition of NaCl, KCl, Ca_2_Cl, MgCl nor any shift in the pH value tested, had any effect on solubility or 2D sheet assembly (Fig 6A and Table 3). In addition simultaneous supplementation of 50 mM arginine and glutamine which were known to effectively act in preventing protein aggregation and precipitation had no influence on sedimentation (Fig 6A lowest panel). Solely by addition of urea at the lowest of 1 M, HP1542 was found shifted to complete solubility (Fig 6A) whereas 2D sheet assembly remained largely unaffected. These results indicate that the observed 2D protein sheets of HP1542 were resistant to a number of treatments.

**Table 3:** Buffer-compositions for HP1542 low spin down assay.

Description	Buffer composition
NaCl	50 mM Tris pH 7,5; 0,5–1,5 M NaCl
KCl	50 mM Tris pH 7,5; 0,5–1,5 M KCl
Urea	50 mM Tris pH 7,5; 50 mM NaCl; 0,5 M Urea
Arg/Glu	50 mM Tris pH 7,5; 50 mM NaCl; 50 mM L-Arg; 50 mM L-Glu
pH 5	50 mM C_2_H_3_NaO_2_, 50 mM NaCl, 5 mM EDTA
pH 6	50 mM MES, 50 mM NaCl, 5mM EDTA
pH 7,5	50 mM Tris, 50 mM NaCl, 5 mM EDTA
pH 8	50 mM Tris, 50 mM NaCl, 5 mM EDTA
pH 9	50 mM Tris, 50 mM NaCl, 5 mM EDTA

Next we aimed at analyzing the functional importance of the N- and C-terminal region of HP1542 concerning their *in vitro* assembly. We produced Strep-HP1542 as N-terminal truncated version beginning with DUF domain (HP1542_112-411_) and as C-terminal truncated version ending with the DUF domain (HP1542_1-339_). Whereas overexpression of HP1542_112-411_ failed due to proteolysis, HP1542_1-339_ could be purified as a soluble protein under standard conditions. Size exclusion chromatography (SEC) using a Superdex 200 Increase 10/300 GL column (GE Heathcare) revealed that HP1542_1-339_ eluted at 11 ml, 14.5 ml and 17 ml corresponding to the size of about 158 kDa, 44kDa and 17 kDa respectively. No HP1542_1-339_ protein was found in the void volume. The monomeric fraction represented the smallest peak. Thus these results demonstrate that HP1542_1-339_ like the entire HP1542 is able to undergo stepwise oligomerization. Next we subjected HP1542_1-339_ to low-spin centrifugation. Just as the entire HP1542 almost same amounts of HP1542_1-339_ were found in freshly purified samples indicating the existence of complete solubility (Fig 6B left upper corner). Subsequently we subjected the purified HP1542_1-339_ to electron microscopy. Interestingly various single filaments of approx. 3 nm widths as well as filament bundles were seen (Fig 6B). Furthermore no clear 2D crystalline arrangement could be observed (Fig 6B inlet). Together, these results suggest that the C-terminal domain of HP1542 is essential for 2D sheet assembly.

### Circular dichroism of purified HP1542 sheets confirms the predominately parallel β-sheet structure

**Fig 7:**
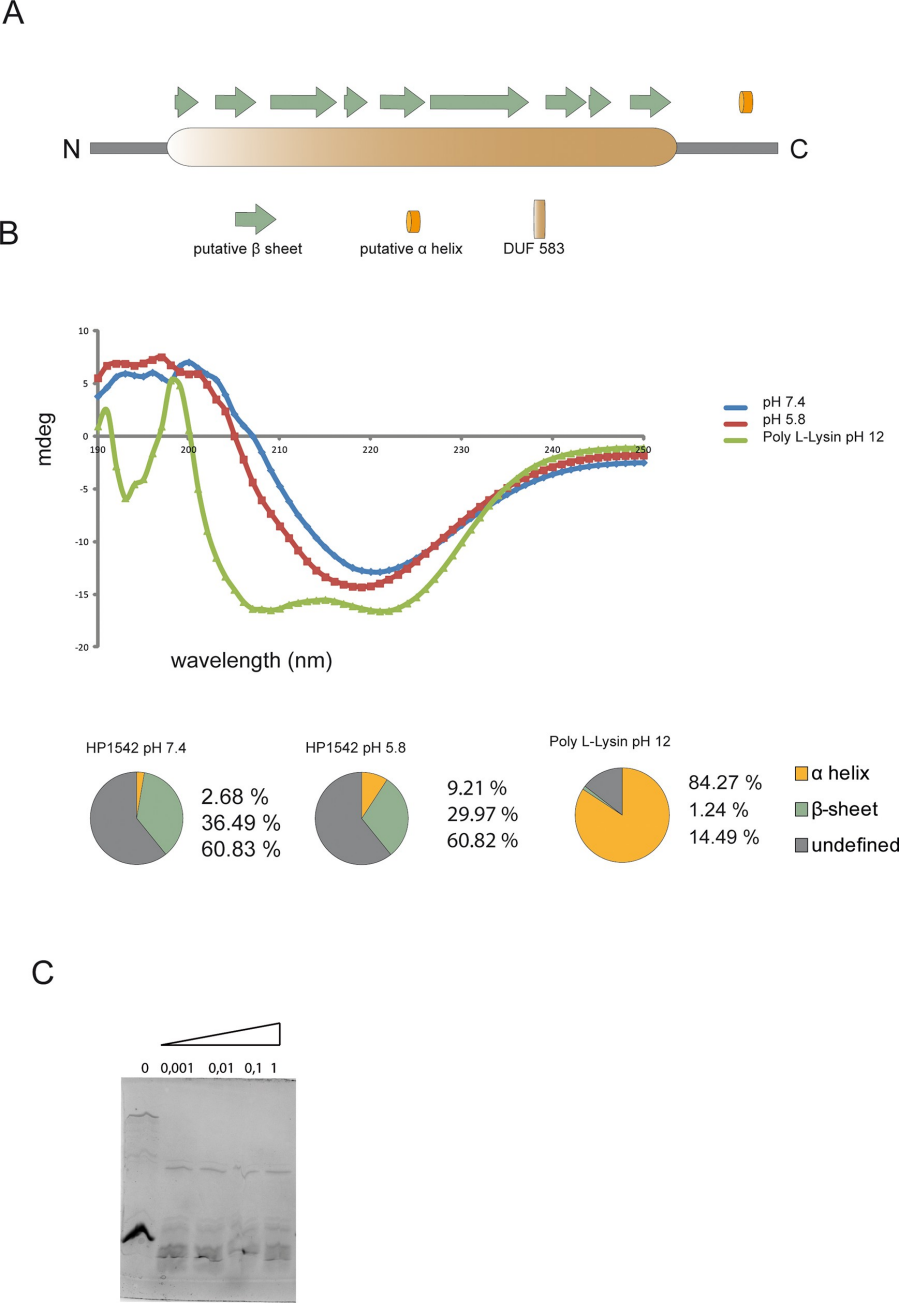
Secondary structure analysis. (A) *In silico* prediction of the secondary structure of HP1542, as created by the PSIPRED server. The Bactofillin domain (DUF583) is illustrated as orange passage, β-sheets as green arrows and α-helices as orange tube. (B) CD spectroscopy of Strep-HP1542. CD-units are plotted in mDeg against the wavelength in 190–250 nm for HP1542 under mild (blue) and acidic (red) conditions as well as for Poly-L-Lysin in pH 12 (green). The calculated secondary structure contributions are illustrated in pie charts. (C) SDS-PAGE analysis of the digestion of Strep-HP1542 with proteinase K (Sigma-Aldrich) at the indicated concentrations (mg ml−1).

By using different protein structure prediction tools like SAS (https://www.ebi.ac.uk/thornton-srv/databases/sas/) (Fig 7A) or AMPHIPASEEK (https://npsa-prabi.ibcp.fr/cgi-bin/npsa_automat.pl?page=/NPSA/npsa_amphipaseek.html), at least one small α-helix at the C-terminus was predicted. In comparison, the pioneer method of protein secondary structure prediction via Chou-Fasman-algorithm resulted in the same amount of α-helices and β-sheets [41], performed on http://capito.nmr.leibniz-fli.de/index.php). Circular dichroism (CD) is an excellent tool for determination of the secondary structure and folding properties of proteins. Thus, we further characterized the structure of the *H*. *pylori* HP1542 bactofilin protein sheets of purified Strep-1542 by circular dichroism (CD). The recorded CD spectrum indicated a minimum at 218 nm (Fig 7B) that is characteristic for a β-sheet-only structure [42] likewise seen for BacM [7]. No clear resemblance to both the control CD spectra of the α-helical references Poly-L-Lysin (Fig 7B) at pH 12 [42] and the CD spectra of Myoglobin were observed. The calculation of percentages of secondary structures was performed using the webtool K2D3 (http://www. ogic.ca/projects/k2d3. [43]). As such, the CD spectra indicated the existence of 2.68% α-helices and 36.49% β-sheets at pH 7.4. At pH 5.8 the amount of α-helices shifted to higher percentages (Fig 7B, lower part). Analyses of the calculated value were validated using the webtools CAPITO [44] (http://capito.nmr.leibniz-fli.de/index.php) and BeStSel (bestsel.elte.hu). In all cases the percentages of α-helices were comparable and β-sheets were most pronounced at pH 7.4.

### HP1542 is not resistant to proteinase K treatment, does not bind thioflavin T and is therefore likely not an amyloid protein

Extended β-helical structures are a characteristic found in amyloid proteins like prion proteins which are associated with severe diseases e.g. the neurodegenerative Alzheimer's disease [45]. However, also a great variety of amyloid proteins with physiological functions such as hormone storage or as structural material are existing [46, 47]. Interestingly it was previously published that the solved ssNMR structure of BacA displayed several similarities to the structure of the prion protein HET-s from the filamentous fungus *Podospora anserina* in its amyloid state [5]. Despite this reported similarity to amyloid proteins, to our knowledge none of the bactofilins analyzed so far were checked for amyloid characteristics. Therefore we set out to analyze whether HP1542 is an amyloid protein. In fact, one of the typical characteristics of the prion proteins is their at least partially resistance to proteinase K treatment in their amyloid conformation [48]. However treatment of HP1542 with proteinase K resulted in complete digestion of the protein (Fig 7C). A further defining characteristic of an amyloid protein is the ability to bind certain dyes such as Congo red and thioflavin T [49]. As such we tested the binding of HP1542 to thioflavin T, but no binding could be observed. However the major biophysical method for testing the typical amyloid features is the x-ray diffraction, defining the existence of the critical 4.7A reflection [50], which is not available for bactofilins so far. Nevertheless despite the tempting hypothesis of HP1542 as a functional amyloid, our results suggest that HP1542 is not an amyloid protein.

### *H*. *pylori* bactofilin proteins from different strains have the same molecular weight and exist as only one variant *in vivo*

The bactofilin of *H*. *pylori* was firstly identified in strain G27 and classified as CcmA homolog of *Proteus mirabilis* [11]. Both CcmA of *P*. *mirabilis* and BacM of *M*. *xanthus* [51] exist in the cells in more than one form. Thus we further analyzed the properties of HP1542 *in vivo*. Therefore we generated a poly- clonal antibody serum against both recombinant HP1542 protein and synthetically synthesized peptide fragments of HP1542 which were further affinity purified (Davids Biotechnology). As negative control we established the HP1542 deletion in strain 26695 analogous to the deletion generated in strain G27 as published [22]. Subsequently anti-HP1542 immunoblots of total cellular proteins of strain 26695 were conducted demonstrating only one single signal at the apparent MW of the full-length HP1542 (S3 Fig). This results was confirmed for several *H*. *pylori* wild type strains like KE, 1061 and G27 (S3 Fig) indicating that HP1542 is not N-terminally truncated like CcmA of *P*. *mirabilis* [11] and BacM of *M*. *xanthus* [51]. Furthermore our results demonstrate that the bactofilin CcmA of *H*. *pylori* strain G27 (G27_HP1480) which was annotated as a protein consisting of 104 amino acids (http://www.uniprot.org/uniprot/B5Z9H3) and thereby would be smaller than the protein HP1542 of strain 26695, has the same size as HP1542. Thus we re-analyzed the DNA sequence of strain G27 and found an alternative ATG start codon at bp position 1607196 of the complement strain [28]. This new start leads to a protein of 136 amino acids which displayed 96.6% sequence identity to HP1542 of strain 26695 analyzed with EMBOSS Needle.

### HP1542 is associated and located close to the cell membrane

**Fig 8:**
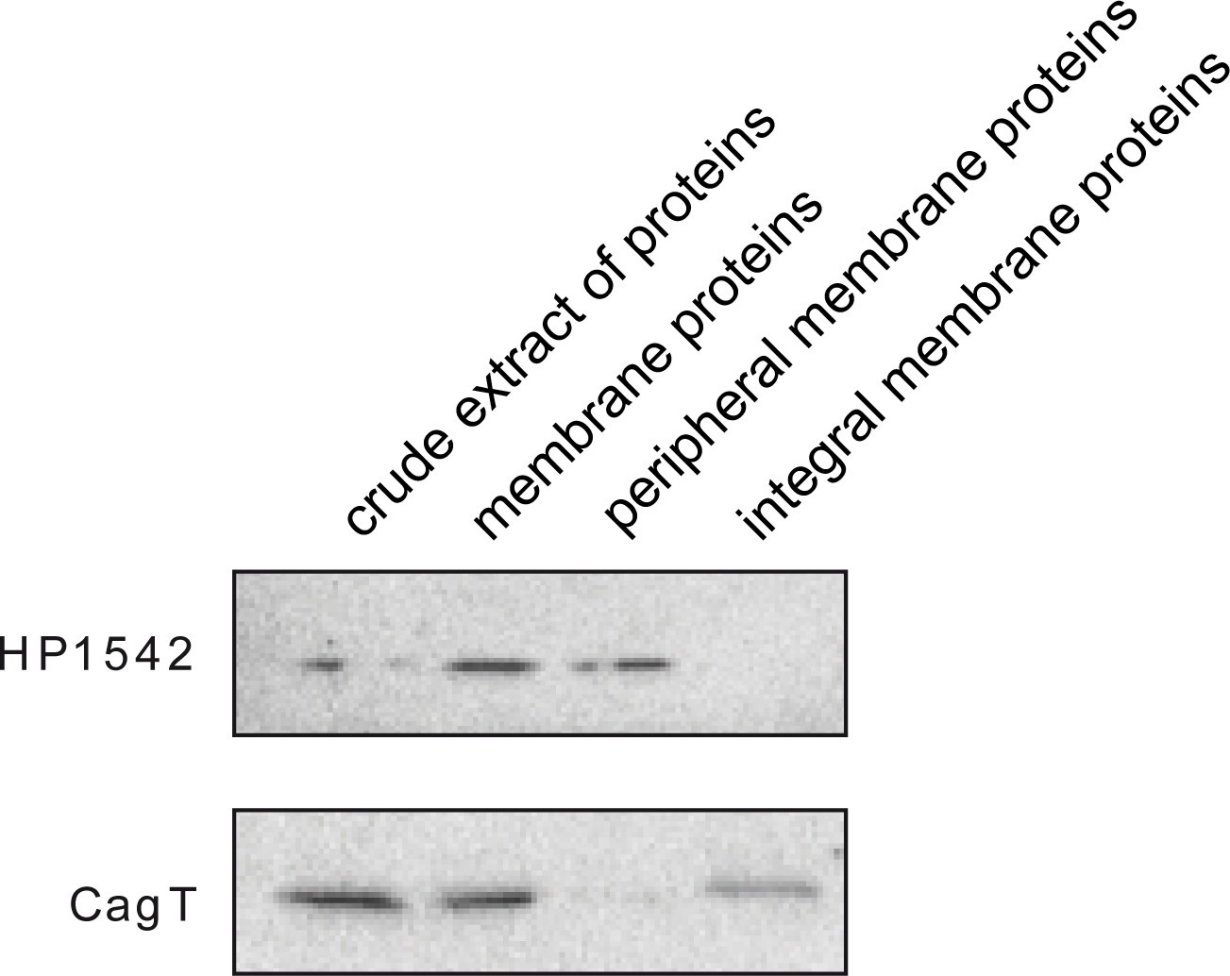
Membrane association of HP1542. Whole-cell lysate of wild-type strain 26695 was fractionated by ultracentrifugation. Samples from the lysate, the soluble fraction, and the insoluble membrane fraction were analyzed by immunoblotting using anti-HP1542 and anti-CagT antiserum. The integral membrane protein CagT serves as control for fractionation efficiency.

To obtain information about the intracellular localization of HP1542, we conducted cell fractionation studies of cell lysates of different *H*. *pylori* wild type strains using ultracentrifugation. All samples were analyzed by immunoblotting using the anti-1542 antiserum. The integral membrane protein CagT [52] served as control for the fractionation efficiency. HP1542 was released in soluble form under alkaline conditions supporting the notion that it is a peripheral membrane protein [53]. Therefore our result confirm that HP1542 like other bactofilins [2] is found to co-sediment with the peripheral membrane proteins [53] indicating that HP1542 is at least partially membrane-associated (Fig 8) and points to an association with the cell envelope.

**Fig 9:**
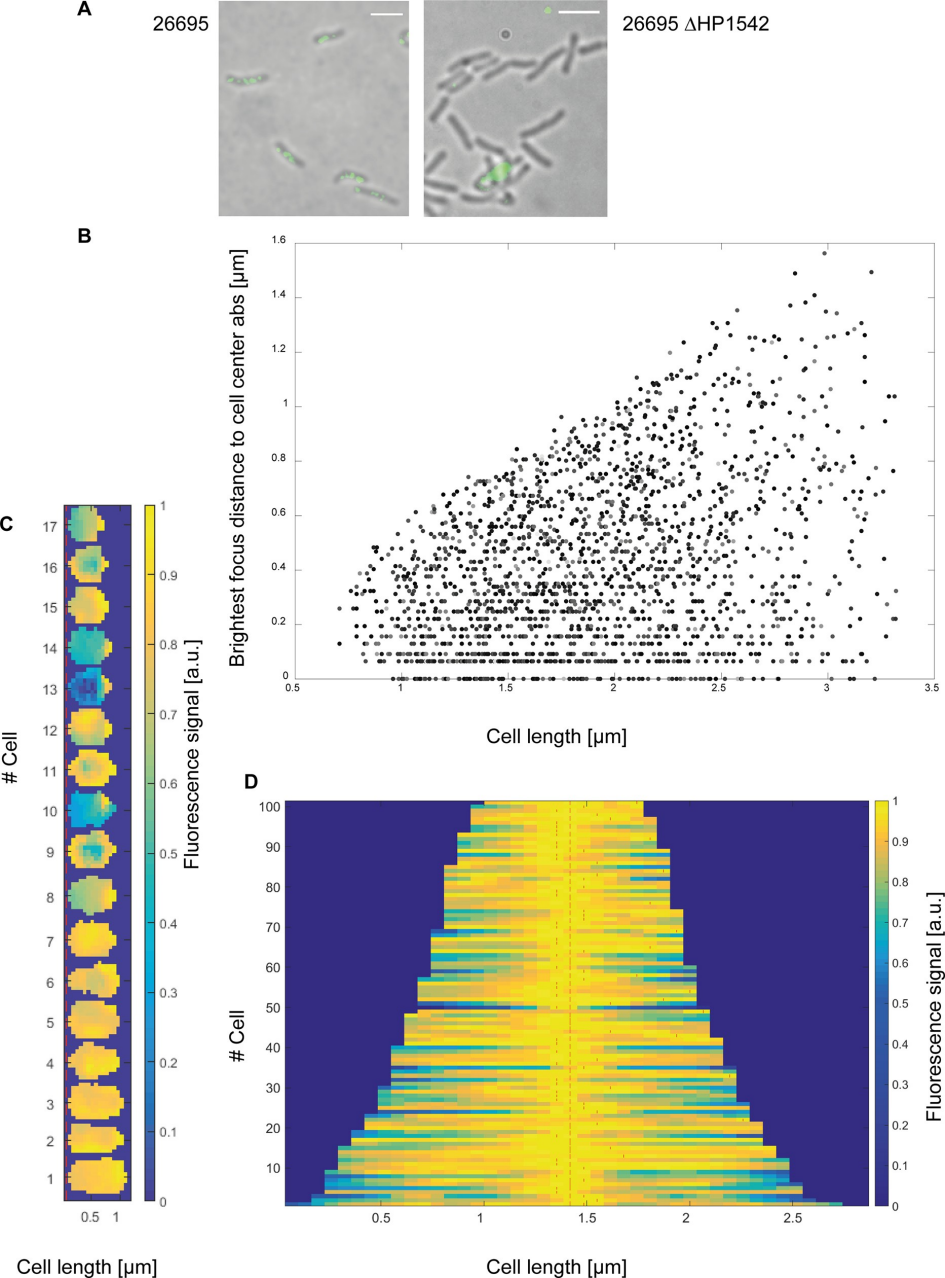
Subcellular localization of HP1542. (A) 26695 wt and 26695 HP1542 deletion mutant were analyzed by immunofluorescence microscopy (IFM) using an anti-HP1542 primary antibody and an Alexa-Fluor 488-conjugated secondary antibody (scale bar 2 μm). (B- D) Quantitative analysis of the brightest spot of all fluorescence signals using BacStalk software. (B) Scatterplot plot depicting the absolute values of the distance of the brightest spot of all fluorescence signals to mid-cell compared to the cell length of the cells. (C) Demograph of small cells below 1 μm. The fluorescence profiles of individual cells were sorted according to cell length with the shortest cell shown at the top and the longest cell shown at the bottom. (D) Demograph of cells with the brightest focus at a defined mid- cell localization (+/- 0.0 64; n = 101) illustrating additional maxima as indicated with red lines.

To gain more information about the localization pattern of HP1542 *in vivo* inside the bacterial cell we performed immunofluorescence (IF) microscopy of liquid-grown bacteria (mid-log phase; growth-dependent high-energy conditions). Antibody specificity was confirmed by using the generated HP1542 deletion strain (Fig 9A). Qualitative analysis of images revealed that under these conditions wt cells generally contained different numbers of patches next to the cell membrane (Fig 9A) both at mid cell and at polar positions. In order to specify the localization pattern in a more detailed and statistically way we used the previously published BacStalk [34] software, which is a MatLab-based software tool for quantitatively analysing images of commonly and uncommonly shaped bacteria. Thereby we analysed the fluorescence signals of 2020 cells. As BacStalk automatically detects and separates cells, all individuals cells were verified by eye resulting in a Gaussian distribution of cell length confirming the mid-log growth phase. Using Balkstalk we measured the distance of the brightest spot of all fluorescence signals to mid-cell and compared this to the cell length of the cells. The measured values were taken as absolute values and thus e.g. a fluorescence signal at mid-cell position is pictured as a value of zero in the resulting scatterplot plot (Fig 9B). Interestingly, no distinct mid-cell localization with a value of zero was found in small cells (below 1 μm). Instead of that small cells were found to exhibit one polar focus or a more or less evenly fluorescence signal distribution (Fig 9C). Examination of cells length in cells with the brightest focus at a defined mid- cell localization (= distance between zero and 0.064 μm; n = 101) showed that 56% had a size between 1 and 1.6 μm, 28% and 16% were 1.7 to 2.3 μm and 2.4 to 2.9 μm in size respectively. However, closer inspection of these cells *via* fluorescence intensity plots revealed that next to the brightest spot at mid-cell location most of the cells displayed additional maxima as indicated by using the BacStalk analysis tool (Fig 9, red lines). With regard to localization of the brightest spot inside the cells, the overall analysis of all cells revealed that in almost two third of the cells (65%) the brightest fluorescence signal was located next to mid-cell (+/- 0.26 μm away from mid-cell) indicating the favor of a mid-cell rather than of a polar localization. As *H*. *pylori* shows an asymmetric cell divisome assembly [33] [23], the results suggests that HP1542 might have a function in cell division as it was suggested for the *S*. *oneidensis* bactofilin (SO_1662) [2].

### Deletion of HP1542 has no influence on resistance to cell wall-targeting antibiotics or on cell wall integrity

In some organism bactofilins may exert their influence over the cell morphology by contributing to proper peptidoglycan (PG) maintenance. As such, in both *M*. *xanthus* and *L*. *biflexa* cells, the absence of bactofilins significantly lowered the tolerance to cell wall-targeting antibiotics [51] [15]. Interestingly the bactofilin deficient mutant of *H*. *pylori* strain G27 was shown to form an altered global peptidoglycan (PG) profile [22], which was similar to those observed for the three LytM peptidoglycan endopeptidase homologues (*csd1*–*3*). Thus an inviting hypothesis is that the bactofilin form a scaffold that is involved in positioning hydrolases which than modify the degree of PG crosslinking only at specific sites [54, 55]. *H*. *pylori* is an important human pathogen of which clinically used antibiotics often target the peptidoglycan biosynthesis enzymes, e.g. Ampicillin and Amoxicillin. *H*. *pylori* produces no β-lactamase homolog and resistance to β-lactam antibiotics is mediated by alterations in the penicillin-binding proteins (PBPs), decreased permeability of the antibiotic into the bacterial cell wall, or a combination of the two strategies. Thus, we tested the influence of HP1542 deletion to ampicillin and amoxicillin treatment *via* ellipsoid- testing. Testing of ampicillin and amoxicillin of both *H*. *pylori* wt 26695 and HP1542 deletion mutant resulted in the MIC of 0.125 μg/μl and 0.06 μg/ml respectively indicating no significant influence on tolerance to these cell wall-targeting antibiotics. However *H*. *pylori* wt is susceptible to both substances making it difficult to interpret whether the HP1542 deletion weakens cell wall stability. Therefore we examined the lysozyme sensitivity of strain 26695 wt compared to its 1542 deletion mutant. Gram-negative bacteria are not generally susceptible to lysozyme because their outer membrane prevents access of the secreted enzyme to the PG layer [35]. Six hours after the treatment with 30 mg/ml lysozyme about 10^5^ viable cells of the wild-type and the HP1542 deletion were counted indicating that the HP1542 mutant is not more sensitive to lysozyme. Cell wall integrity was further assessed by comparing growth of *H*. *pylori* wt vs HP1542 deletion strain in hyper-osmotic conditions (10 g/l NaCl) to the standard BBF formulation containing 5 g/l NaCl. Both strains grew to similar high densities in standard media (OD600 2.4+/- 0.04 and 2.4+/- 0.08 for wt and 1542 mutant respectively) and in hyper-osmotic media (OD600 2.6+/- 0.05 and 2.4+/- 0.05 for wt and 1542 mutant respectively). Together, the antibiotic and osmotic sensitives of the HP1542 mutant indicate that this bactofilin is not contributing to cell wall stability.

## Discussion

By now it is accepted that microorganisms also have well-organized cellular interiors [1] including bacterial cytoskeleton proteins participating in many important physiological functions. Among these, bactofilins are a widespread group of proteins exclusively found in bacteria. In *H*. *pylori*, which is an important pathogenic organism, the bactofilin homolog has not been biochemically characterized to date indicating a definite paucity of information. Our work provides the first insight into the properties of *H*. *pylori* reference strain 26695 bactofilin homolog HP1542 in terms of protein polymerization, protein stability and aggregation. Furthermore we supplemented our *in vitro* data with localization studies using zonal centrifugation and immunofluorescence and growth experiments considering cell wall integrity.

In contrast to other bactofilin proteins HP1542 could be purified as a soluble monomeric protein which subsequently polymerized spontaneously into predominant 2-D sheets. Importantly 2-D sheet formation was observed to be influenced by the protein tag attached to the protein as sheet formation could rarely be found in purified fractions of His_6_-1542. Furthermore truncation of the C-terminus resulted in soluble polymeric fibers and bundles of filaments and the complete loss of the 2-D sheets. Electron microscopy analysis of purified *H*. *pylori* bactofilin without tag clearly demonstrated the authenticity of the sheet-like arrangement. Likewise Vasa *et al*. reported that purified fractions of *C*. *crescentus* BacA additionally contained sheet-like 2-D crystalline arrays [5]. Thereby and together with the results from electron cryotomographic studies of *C*. *crescentus* it was suggested that these 2-D crystalline arrays might represent the actual arrangement of BacA polymers in the native context. In addition HP1542 protofilaments of about 3 nm in width comparable to those reported for BacA of *C*. *cresecntus* and BacM of *M*. *xanthus* were seen next to the crystalline arrays [2, 7]. The complete loss of 2-D arrays in the C-terminal truncated version of HP1542 clearly demonstrates that the C-terminus of HP1542 is essential for sheet assembly but dispensable for filament polymerization. Likewise the C-terminus of BacM contributed to the stability of the fiber, and the lateral packing of filaments. (Zuckerman). As such a plausible way of HP1542 2-D sheet generation is that the *H*. *pylori* bactofilin firstly built up protofilaments, which than further self-assemble with contribution of the C-termini into 2-D sheets. However there will be further studies needed to clarify the way of contribution of the C-terminus as well as to analyze its role *in vivo* inside the bacterial cell. Unfortunately, neither time nor buffer condition influencing the polymerization process of HP1542 could be identified. While this result is per se unsatisfactory, it is in line within the literature demonstrating the robustness of bactofilins, e.g. as observed for *M*. *xanthus* BacM [7]. However unlike BacM the polymerization of HP1542 was not influenced by pH values and does also polymerize at any NaCl concentration tested. Furthermore addition of 20 mM glycin, which disrupted the lateral interactions of BacM [7] conducted no obvious change within the 2-D crystalline structures. In *M*. *xanthus* BacM polymerization even occurred in the presence of chaotropic agents such as one molar urea. Addition of one molar urea to polymerized HP1542 resulted in the restoration of complete solubility despite remaining 2-D assemblies as seen in electron microscopy. Next, atomic force microscopy analysis confirmed the existence of extended crystalline arrays. Interestingly all 2-D structures measured had an average height of about 5 nm suggesting a single or double protein layer rather than a multilayer. In our hands it was not possible to achieve nanometer resolution as protein subunits seem to spread upon contact *via* tapping mode with the cantilever at least at the present stage. Thus despite the extraordinary stability of 2-D structures in terms of different environmental conditions, the lateral stability according to physical pressure during the atomic force microscopy measurements is weak. Further experiments are required to characterize the mechanical properties of these amazing 2-D crystalline arrays. In addition symmetry of proteins structures is an important feature that has been associated with protein function, evolution and stability [56]. The HP1542 2-D structures observed *via* electron microscopy gave the impression of a periodical arrangement or a lattice. Using the software package ANIMETRA CRYSTALS, which acts via discrete Fourier transformation, we were able to define that the protein sheet most likely exhibits simple p2 symmetry. Taking into account that the DUF583 domain of bactofilins exhibit a triangular hydrophobic core structure [5, 7], p2 symmetry results in an oblique lattice composed of scalene triangles. Analysis of Circular dichroism spectra of the purified *H*. *pylori* bactofilin confirmed the prominent β-helical secondary structure of HP1542, which was most pronounced in neutral pH.

In previous studies it was reported that the DUF583 domain assembles in a β-helical fold, which has not been reported by any cytoskeletal filament protein so far. Nevertheless, the motif itself was known before and firstly found in the bacterial pectate lyase in 1993 [57, 58]. Interestingly it was also reported that the solved ssNMR structure of *C*. *crescentus* BacA bears structural similarities to that of the fungal prion protein HET-s in its amyloid state [5]. The HET-s fibril is a left-handed β-solenoid with each protein molecule forming two helical turns [10] by which multilayer formation of two layers was reported for HET-s(218–289). In addition synthetically engineered β-solenoid proteins were shown to be extraordinarily stable toward organic solvents, urea, and pH extremes and temperature [59]. However despite sharing some structural and biochemical properties with amyloid proteins, the HP1542 bactofilin was neither resistant to proteinase K digestion nor stainable with thioflavin T which is often used for identifying amyloid fibrils both *in vivo* and *in vitro* [60]. Thus our data suggest that the *H*. pylori bactofilin does not belong to the family of amyloid proteins.

Biology of bactofilins is still largely unexplored [4]. In *H*. *pylori* the bactofilin homolog was identified as homolog of CcmA of *P*. *mirabilis* [22]. In this previous study *H*. *pylori* cells lacking the bactofilin were shown to lose their helical shape and increase the peptide crosslinking in the peptidoglycan sacculus. Likewise cell morphology of *P*. *mirabilis* and *M*. *xanthus* were affected by the loss of bactofilins [51] [11]. Intriguingly, both CcmA and BacM exist in a full-length and an N-terminally truncated form. Contrarily, our results demonstrate that *H*. *pylori* contain only one variant of its bactofilin *in vivo*. Interestingly, despite the known heterogeneity of *H*. *pylori* strains, all bactofilin proteins analyzed from different strains displayed the same molecular weight. Moreover this was also true for the bactofilin protein CcmA of *H*. *pylori* strain G27 (G27_HP1480) which was annotated as 104 amino acid protein before [28]. Re-analyzing the DNA sequence revealed an alternative start resulting in a 136 amino acid protein thereby confirming the identical size of *H*. *pylori* bactofilins.

Cell fractionation experiments revealed that HP1542 partitions with the membrane-associated proteins as do bactofilins in *L*. *biflexa*. *C*. *crescentus* and *P*. *mirabilis* [2] [11] [15]. Previous results supported the connections between PG synthesis, cell shape and intermediate filament proteins in curved organisms [55] [51] [61]. As such the localization of a peptidoglycan synthase involved in pole morphogenesis [2] was influenced by the two bactofilin paralogs in *C*. *crescentus*. In *L*. *biflexa* one bactofilin mutant displayed a significantly impaired ability to cope with both osmotic stress and cell wall-targeting antibiotics. In addition, absence of BacM leads to an increased sensitivity to antibiotics targeting cell wall biosynthesis [51]. In a previous study it has been shown *tha*t *H*. *pylori* cells devoid of the bactofilin homolog displayed an increase in peptide crosslinking in the peptidoglycan sacculus [22]. However in contrast to LbbD and BacM deficient mutants of *L*. *biflexa* and *M*. *xanthus* respectively, in *H*. *pylori* absence of HP1542 did not increase the sensitivity to antibiotics targeting cell wall antibiotics. Both the bactofilin deficient mutant and the *H*. *pylori* wt showed identical antimicrobial susceptibility for two beta-lactam antibiotics. Additionally, lysozyme treatment resulted in the same amount of survived cells indicating the deletion of HP1542 did not weaken cell wall integrity. Likewise the ability to cope with osmotic stress was the same as both *H*. *pylori* wt and the HP1542 deletion mutant grew to similar high densities in hyper-osmotic conditions. Interestingly, a previous study tested the sensitivity to antimicrobial peptides, acid, and medically administered amoxicillin in the *H*. *pylori* peptidoglycan hydolase Csd1 deficient mutant [22]. This mutant exhibited similar alterations in its peptidoglycan layer as the bactofilin homolog (and as two other LytM peptidoglycan endopeptidases), and it was also stated that cell wall changes produced in these mutants do not appreciably alter cell wall integrity. Nevertheless it is an attractive hypothesis that the morphogenetic effect of bactofilins is generally achieved through the interaction with proteins involved in PG synthesis. In support of this, it was shown recently that the non-enzymatic Csd5 protein of *H*. *pylori strain* G27, which also contributes to the helical shape *of H*. *pylori*, interact with the bactofilin as well as with a PG precursor synthesis enzyme and the ATP synthase [55]. In this study the existence of a shape promoting protein complex that spans the cytoplasmic membrane and may connect the cell wall in the periplasm with the cytoplasmic cytoskeleton was suggested.

Subcellular localization is one of the main aspects defining protein function. Both epi- and immuno- fluorescence microscopy contributed to the analysis of the intracellular localization of bactofilins. Interestingly, different subcellular distributions of bactofilins were observed in different bacterial species, suggesting that they have adopted a range of different cellular functions. As such BacA and BacB from *C*. *crescentus* form a cluster at the stalked cell pole [2] mediating localization of peptidoglycan synthase. Immunofluorescence microscopy showed that BacM of *M*. *xanthus*, which is important for proper cell shape maintenance, has a variable localization pattern forming either helical cables that extend throughout the cell or rod-like filaments originating at the cell poles [51]. In addition the three bactofilins BacNOP of *M*. *xanthus* co-assemble into extended scaffolds at the cell poles thereby controlling the positioning of the chromosomal origin segregation machinery [4]. The localization pattern of *S*. *oneidensis* bactofilin SO1662 is reminiscent of FtsZ localization and therefore clearly points to a role in cell division [2]. In *H*. *pylori* immunofluorescence analysis demonstrated that HP1542 localized as subcellular assemblies in different numbers next to the cell membrane both at mid cell and at polar positions. Using the previously published BacStalk [34] software, the quantitative statistical analysis revealed that the brightest fluorescence signal in most cells (65%) was located next to mid-cell indicating the favor of mid-cell rather than polar localization. However the average value of this localization was 0.26 μm whereas a clear mid-cell localization would be depicted as value zero. Taking into account that *H*. *pylori* shows an asymmetric cell division assembly [33], these results suggest a contribution in cell division. Furthermore small cells (<1 μm) usually exhibited one polar focus or a more or less evenly fluorescence signal distribution resembling FtsZ localization in *H*. *pylori* [33] [23]. Interestingly *H*. *pylori* shows unique cell division profile with polar peptidoglycan synthesis in non-dividing cells and both polar and mid-cell peptidoglycan synthesis in dividing cells [33]. Bacterial cell division and elongation has been studied primarily in *E*. *coli* and *B*. *subtilis* and carried out by two large and highly dynamic molecular machines, known as the divisome and the elongasome, which both contribute to peptidoglycan synthesis [62]. At present, we cannot explain how bactofilins may contribute to cell morphology and whether the bactofilin in *H*. *pylori* is part of the divisom or the elongasome complex or both. Nevertheless the results presented here provide a platform for understanding the intimate co-evolution of morphology and cell division in the human pathogen *H*. *pylori*.

## Supporting information

S1 Fig ESI-TOF/MS analysis of affinity purified Strep-HP1542. ESI-TOF/MS analysis of affinity purified Strep-HP1542 deconvolated electronspray mass spectrum of affinity purified HP1542, given in relative Intensity (%) against molecular weight (m/z).
(TIF)

S2 Fig Comparison of HP1542 to BacA of *C. crescentus*. Comparison of HP1542 (NCBI Reference Sequence: NP_208333.1) to BacA (UniProtKB—Q9A753 (Q9A753_CAUVC)) in terms of primary sequence, secondary structure motifs and the DUF 583 domains as indicated. Conserved residues are marked with asterisks.
(TIF)

S3 Fig Immunoblot analysis of bactofilin proteins in different *H. pylori strains*. Western blot using HP1542 specific antiserum, strains as indicated above the lanes (wt, wild type; Δ1542, HP1542 deletion strain). Equal amounts of protein were loaded onto each lane.
(TIF)

## References

[1] Cho H. The role of cytoskeletal elements in shaping bacterial cells. Journal of microbiology and biotechnology. 2015;25(3):307–16. Epub 2014/09/30. . 25262683 10.4014/jmb.1409.09047

[2] Kuhn J, Briegel A, Morschel E, Kahnt J, Leser K, Wick S, et al Bactofilins, a ubiquitous class of cytoskeletal proteins mediating polar localization of a cell wall synthase in *Caulobacter crescentus*. The EMBO journal. 2010;29(2):327–39. Epub 2009/12/05. 10.1038/emboj.2009.358 19959992 PMC2824468

[3] Marchler-Bauer A, Anderson JB, Chitsaz F, Derbyshire MK, DeWeese-Scott C, Fong JH, et al CDD: specific functional annotation with the Conserved Domain Database. Nucleic acids research. 2009;37(Database issue):D205–10. Epub 2008/11/06. 10.1093/nar/gkn845 18984618 PMC2686570

[4] Lin L, Thanbichler M. Nucleotide-independent cytoskeletal scaffolds in bacteria. Cytoskeleton (Hoboken, NJ). 2013;70(8):409–23. Epub 2013/07/16. 10.1002/cm.21126 . 23852773

[5] Vasa S, Lin L, Shi C, Habenstein B, Riedel D, Kuhn J, et al beta-Helical architecture of cytoskeletal bactofilin filaments revealed by solid-state NMR. Proceedings of the National Academy of Sciences of the United States of America. 2015;112(2):E127–36. Epub 2015/01/01. 10.1073/pnas.1418450112 25550503 PMC4299214

[6] Shi C, Fricke P, Lin L, Chevelkov V, Wegstroth M, Giller K, et al Atomic-resolution structure of cytoskeletal bactofilin by solid-state NMR. Science advances. 2015;1(11):e1501087 Epub 2015/12/15. 10.1126/sciadv.1501087 26665178 PMC4672760

[7] Zuckerman DM, Boucher LE, Xie K, Engelhardt H, Bosch J, Hoiczyk E. The bactofilin cytoskeleton protein BacM of *Myxococcus xanthus* forms an extended beta-sheet structure likely mediated by hydrophobic interactions. PloS one. 2015;10(3):e0121074 Epub 2015/03/25. 10.1371/journal.pone.0121074 25803609 PMC4372379

[8] Kassem MM, Wang Y, Boomsma W, Lindorff-Larsen K. Structure of the Bacterial Cytoskeleton Protein Bactofilin by NMR Chemical Shifts and Sequence Variation. Biophysical journal. 2016;110(11):2342–8. Epub 2016/06/09. 10.1016/j.bpj.2016.04.039 27276252 PMC4922582

[9] Flores-Fernandez JM, Rathod V, Wille H. Comparing the Folds of Prions and Other Pathogenic Amyloids. Pathogens (Basel, Switzerland). 2018;7(2). Epub 2018/05/08. 10.3390/pathogens7020050 29734684 PMC6027354

[10] Wasmer C, Lange A, Van Melckebeke H, Siemer AB, Riek R, Meier BH. Amyloid fibrils of the HET-s(218–289) prion form a beta solenoid with a triangular hydrophobic core. Science (New York, NY). 2008;319(5869):1523–6. Epub 2008/03/15. 10.1126/science.1151839 . 18339938

[11] Hay NA, Tipper DJ, Gygi D, Hughes C. A novel membrane protein influencing cell shape and multicellular swarming of Proteus mirabilis. Journal of bacteriology. 1999;181(7):2008–16. Epub 1999/03/27. 10094676 10.1128/jb.181.7.2008-2016.1999 PMC93611

[12] Bulyha I, Lindow S, Lin L, Bolte K, Wuichet K, Kahnt J, et al Two small GTPases act in concert with the bactofilin cytoskeleton to regulate dynamic bacterial cell polarity. Developmental cell. 2013;25(2):119–31. Epub 2013/04/16. 10.1016/j.devcel.2013.02.017 . 23583757

[13] Lin L, Osorio Valeriano M, Harms A, Sogaard-Andersen L, Thanbichler M. Bactofilin-mediated organization of the ParABS chromosome segregation system in *Myxococcus xanthus*. Nature communications. 2017;8(1):1817 Epub 2017/11/29. 10.1038/s41467-017-02015-z 29180656 PMC5703909

[14] El Andari J, Altegoer F, Bange G, Graumann PL. *Bacillus subtilis* Bactofilins Are Essential for Flagellar Hook- and Filament Assembly and Dynamically Localize into Structures of Less than 100 nm Diameter underneath the Cell Membrane. PloS one. 2015;10(10):e0141546 Epub 2015/10/31. 10.1371/journal.pone.0141546 26517549 PMC4627819

[15] Jackson KM, Schwartz C, Wachter J, Rosa PA, Stewart PE. A widely conserved bacterial cytoskeletal component influences unique helical shape and motility of the spirochete *Leptospira biflexa*. Molecular microbiology. 2018;108(1):77–89. Epub 2018/01/25. 10.1111/mmi.13917 29363884 PMC5867249

[16] Marshall BJ, Warren JR. Unidentified curved bacilli in the stomach of patients with gastritis and peptic ulceration. Lancet (London, England). 1984;1(8390):1311–5. Epub 1984/06/16. . 6145023 10.1016/s0140-6736(84)91816-6

[17] Blaser MJ. *Helicobacter pylori* and the pathogenesis of gastroduodenal inflammation. The Journal of infectious diseases. 1990;161(4):626–33. Epub 1990/04/01. 10.1093/infdis/161.4.626 . 2181029

[18] Parsonnet J. Gastric adenocarcinoma and *Helicobacter pylori* infection. The Western journal of medicine. 1994;161(1):60 Epub 1994/07/01. 7941511 PMC1011373

[19] Robinson K, Letley DP, Kaneko K. The Human Stomach in Health and Disease: Infection Strategies by *Helicobacter pylori*. Current topics in microbiology and immunology. 2017;400:1–26. Epub 2017/01/27. 10.1007/978-3-319-50520-6_1 . 28124147

[20] Hazell SL, Lee A, Brady L, Hennessy W. *Campylobacter pyloridis* and gastritis: association with intercellular spaces and adaptation to an environment of mucus as important factors in colonization of the gastric epithelium. The Journal of infectious diseases. 1986;153(4):658–63. Epub 1986/04/01. 10.1093/infdis/153.4.658 . 3950447

[21] Worku ML, Sidebotham RL, Walker MM, Keshavarz T, Karim QN. The relationship between *Helicobacter pylori* motility, morphology and phase of growth: implications for gastric colonization and pathology. Microbiology (Reading, England). 1999;145 (Pt 10):2803–11. Epub 1999/10/28. 10.1099/00221287-145-10-2803 . 10537202

[22] Sycuro LK, Pincus Z, Gutierrez KD, Biboy J, Stern CA, Vollmer W, et al Peptidoglycan crosslinking relaxation promotes *Helicobacter pylori's* helical shape and stomach colonization. Cell. 2010;141(5):822–33. Epub 2010/06/01. 10.1016/j.cell.2010.03.046 20510929 PMC2920535

[23] Specht M, Schatzle S, Graumann PL, Waidner B. *Helicobacter pylori* possesses four coiled-coil-rich proteins that form extended filamentous structures and control cell shape and motility. Journal of bacteriology. 2011;193(17):4523–30. Epub 2011/06/07. 10.1128/JB.00231-11 21642462 PMC3165534

[24] Krzyzek P, Gosciniak G. Morphology of *Helicobacter pylori* as a result of peptidoglycan and cytoskeleton rearrangements. Przeglad gastroenterologiczny. 2018;13(3):182–95. Epub 2018/10/12. 10.5114/pg.2018.78284 30302161 PMC6173076

[25] Waidner B, Specht M, Dempwolff F, Haeberer K, Schaetzle S, Speth V, et al A novel system of cytoskeletal elements in the human pathogen *Helicobacter pylori*. PLoS pathogens. 2009;5(11):e1000669 Epub 2009/11/26. 10.1371/journal.ppat.1000669 19936218 PMC2776988

[26] Schatzle S, Specht M, Waidner B. Coiled coil rich proteins (Ccrp) influence molecular pathogenicity of *Helicobacter pylori*. PloS one. 2015;10(3):e0121463 Epub 2015/03/31. 10.1371/journal.pone.0121463 25822999 PMC4379086

[27] Tomb JF, White O, Kerlavage AR, Clayton RA, Sutton GG, Fleischmann RD, et al The complete genome sequence of the gastric pathogen *Helicobacter pylori*. Nature. 1997;388(6642):539–47. Epub 1997/08/07. 10.1038/41483 . 9252185

[28] Baltrus DA, Amieva MR, Covacci A, Lowe TM, Merrell DS, Ottemann KM, et al The complete genome sequence of *Helicobacter pylori* strain G27. Journal of bacteriology. 2009;191(1):447–8. Epub 2008/10/28. 10.1128/JB.01416-08 18952803 PMC2612421

[29] Goodwin A, Kersulyte D, Sisson G, Veldhuyzen van Zanten SJ, Berg DE, Hoffman PS. Metronidazole resistance in *Helicobacter pylori* is due to null mutations in a gene (rdxA) that encodes an oxygen-insensitive NADPH nitroreductase. Molecular microbiology. 1998;28(2):383–93. Epub 1998/06/11. . 9622362 10.1046/j.1365-2958.1998.00806.x

[30] Sarkar G, Sommer SS. The "megaprimer" method of site-directed mutagenesis. BioTechniques. 1990;8(4):404–7. Epub 1990/04/01. . 2340178

[31] Ho SN, Hunt HD, Horton RM, Pullen JK, Pease LR. Site-directed mutagenesis by overlap extension using the polymerase chain reaction. Gene. 1989;77(1):51–9. Epub 1989/04/15. . 2744487 10.1016/0378-1119(89)90358-2

[32] Leying H, Suerbaum S, Geis G, Haas R. Cloning and genetic characterization of a *Helicobacter pylori* flagellin gene. Molecular microbiology. 1992;6(19):2863–74. Epub 1992/10/01. . 1435261 10.1111/j.1365-2958.1992.tb01466.x

[33] Kamran M, Dubey P, Verma V, Dasgupta S, Dhar SK. *Helicobacter pylori* shows asymmetric and polar cell divisome assembly associated with DNA replisome. The FEBS journal. 2018;285(13):2531–47. Epub 2018/05/11. 10.1111/febs.14499 . 29745002

[34] Hartmann R, van Teeseling MCF, Thanbichler M, Drescher K. BacStalk: a comprehensive and interactive image analysis software tool for bacterial cell biology. bioRxiv. 2018:360230 10.1101/360230 32190923

[35] Wang G, Lo LF, Forsberg LS, Maier RJ. *Helicobacter pylori* peptidoglycan modifications confer lysozyme resistance and contribute to survival in the host. mBio. 2012;3(6):e00409–12. Epub 2012/12/12. 10.1128/mBio.00409-12 23221800 PMC3517862

[36] Gancz H, Jones KR, Merrell DS. Sodium chloride affects *Helicobacter pylori* growth and gene expression. Journal of bacteriology. 2008;190(11):4100–5. Epub 2008/04/01. 10.1128/JB.01728-07 18375562 PMC2395038

[37] An DR, Im HN, Jang JY, Kim HS, Kim J, Yoon HJ, et al Structural Basis of the Heterodimer Formation between Cell Shape-Determining Proteins Csd1 and Csd2 from *Helicobacter pylori*. PloS one. 2016;11(10):e0164243 Epub 2016/10/07. 10.1371/journal.pone.0164243 27711177 PMC5053510

[38] Okumura N, Tamura J, Takao T. Evidence for an essential role of intradimer interaction in catalytic function of carnosine dipeptidase II using electrospray-ionization mass spectrometry. Protein science: a publication of the Protein Society. 2016;25(2):511–22. Epub 2015/11/10. 10.1002/pro.2842 26549037 PMC4815345

[39] Herrmann H, Aebi U. Intermediate filaments: molecular structure, assembly mechanism, and integration into functionally distinct intracellular Scaffolds. Annual review of biochemistry. 2004;73:749–89. Epub 2004/06/11. 10.1146/annurev.biochem.73.011303.073823 . 15189158

[40] Stingl K, Altendorf K, Bakker EP. Acid survival of *Helicobacter pylori*: how does urease activity trigger cytoplasmic pH homeostasis? Trends in microbiology. 2002;10(2):70–4. Epub 2002/02/06. . 11827807 10.1016/s0966-842x(01)02287-9

[41] Chou PY, Fasman GD. Prediction of protein conformation. Biochemistry. 1974;13(2):222–45. Epub 1974/01/15. 10.1021/bi00699a002 . 4358940

[42] Greenfield NJ. Using circular dichroism spectra to estimate protein secondary structure. Nature protocols. 2006;1(6):2876–90. Epub 2007/04/05. 10.1038/nprot.2006.202 17406547 PMC2728378

[43] Louis-Jeune C, Andrade-Navarro MA, Perez-Iratxeta C. Prediction of protein secondary structure from circular dichroism using theoretically derived spectra. Proteins. 2012;80(2):374–81. Epub 2011/11/19. 10.1002/prot.23188 . 22095872

[44] Wiedemann C, Bellstedt P, Gorlach M. CAPITO—a web server-based analysis and plotting tool for circular dichroism data. Bioinformatics (Oxford, England). 2013;29(14):1750–7. Epub 2013/05/18. 10.1093/bioinformatics/btt278 . 23681122

[45] Kelly JW. Alternative conformations of amyloidogenic proteins govern their behavior. Current opinion in structural biology. 1996;6(1):11–7. Epub 1996/02/01. . 8696966 10.1016/s0959-440x(96)80089-3

[46] Greenwald J, Riek R. Biology of amyloid: structure, function, and regulation. Structure (London, England: 1993). 2010;18(10):1244–60. Epub 2010/10/16. 10.1016/j.str.2010.08.009 . 20947013

[47] DePas WH, Chapman MR. Microbial manipulation of the amyloid fold. Research in microbiology. 2012;163(9–10):592–606. Epub 2012/10/31. 10.1016/j.resmic.2012.10.009 23108148 PMC3532741

[48] Kocisko DA, Priola SA, Raymond GJ, Chesebro B, Lansbury PT Jr, Caughey B. Species specificity in the cell-free conversion of prion protein to protease-resistant forms: a model for the scrapie species barrier. Proceedings of the National Academy of Sciences of the United States of America. 1995;92(9):3923–7. Epub 1995/04/25. 10.1073/pnas.92.9.3923 7732006 PMC42074

[49] Elghetany MT, Saleem A. Methods for staining amyloid in tissues: a review. Stain technology. 1988;63(4):201–12. Epub 1988/07/01. . 2464206 10.3109/10520298809107185

[50] Jahn TR, Makin OS, Morris KL, Marshall KE, Tian P, Sikorski P, et al The common architecture of cross-beta amyloid. Journal of molecular biology. 2010;395(4):717–27. Epub 2009/09/29. 10.1016/j.jmb.2009.09.039 . 19781557

[51] Koch MK, McHugh CA, Hoiczyk E. BacM, an N-terminally processed bactofilin of *Myxococcus xanthus*, is crucial for proper cell shape. Molecular microbiology. 2011;80(4):1031–51. Epub 2011/03/19. 10.1111/j.1365-2958.2011.07629.x 21414039 PMC3091990

[52] Frick-Cheng AE, Pyburn TM, Voss BJ, McDonald WH, Ohi MD, Cover TL. Molecular and Structural Analysis of the *Helicobacter pylori* cag Type IV Secretion System Core Complex. mBio. 2016;7(1):e02001–15. Epub 2016/01/14. 10.1128/mBio.02001-15 26758182 PMC4725015

[53] Fujiki Y, Hubbard AL, Fowler S, Lazarow PB. Isolation of intracellular membranes by means of sodium carbonate treatment: application to endoplasmic reticulum. The Journal of cell biology. 1982;93(1):97–102. Epub 1982/04/01. 10.1083/jcb.93.1.97 7068762 PMC2112113

[54] van Teeseling MCF, de Pedro MA, Cava F. Determinants of Bacterial Morphology: From Fundamentals to Possibilities for Antimicrobial Targeting. Frontiers in microbiology. 2017;8:1264 Epub 2017/07/26. 10.3389/fmicb.2017.01264 28740487 PMC5502672

[55] Blair KM, Mears KS, Taylor JA, Fero J, Jones LA, Gafken PR, et al The *Helicobacter pylori* cell shape promoting protein Csd5 interacts with the cell wall, MurF, and the bacterial cytoskeleton. Molecular microbiology. 2018;110(1):114–27. Epub 2018/07/25. 10.1111/mmi.14087 30039535 PMC6195823

[56] Goodsell DS, Olson AJ. Structural symmetry and protein function. Annual review of biophysics and biomolecular structure. 2000;29:105–53. Epub 2000/08/15. 10.1146/annurev.biophys.29.1.105 . 10940245

[57] Yoder MD, Lietzke SE, Jurnak F. Unusual structural features in the parallel beta-helix in pectate lyases. Structure (London, England: 1993). 1993;1(4):241–51. Epub 1993/12/15. . 8081738 10.1016/0969-2126(93)90013-7

[58] Kajava AV, Squire JM, Parry DA. Beta-structures in fibrous proteins. Advances in protein chemistry. 2006;73:1–15. Epub 2006/12/28. 10.1016/S0065-3233(06)73001-7 . 17190609

[59] Peng Z, Peralta MDR, Toney MD. Extraordinarily Stable Amyloid Fibrils Engineered from Structurally Defined beta-Solenoid Proteins. Biochemistry. 2017;56(45):6041–50. Epub 2017/10/25. 10.1021/acs.biochem.7b00364 . 29064686

[60] Rovnyagina NR, Sluchanko NN, Tikhonova TN, Fadeev VV, Litskevich AY, Maskevich AA, et al Binding of thioflavin T by albumins: An underestimated role of protein oligomeric heterogeneity. International journal of biological macromolecules. 2018;108:284–90. Epub 2017/12/07. 10.1016/j.ijbiomac.2017.12.002 . 29208556

[61] Ausmees N, Kuhn JR, Jacobs-Wagner C. The bacterial cytoskeleton: an intermediate filament-like function in cell shape. Cell. 2003;115(6):705–13. Epub 2003/12/17. . 14675535 10.1016/s0092-8674(03)00935-8

[62] Szwedziak P, Lowe J. Do the divisome and elongasome share a common evolutionary past? Current opinion in microbiology. 2013;16(6):745–51. Epub 2013/10/08. 10.1016/j.mib.2013.09.003 . 24094808

