# Supplementary figures and images for: Biochemical characterization of the Helicobacter pylori bactofilin-homolog HP1542
Source: PLoS One. 2019 Jun 24;14(6):e0218474. doi: 10.1371/journal.pone.0218474 (PMC6590870; doi:10.1371/journal.pone.0218474)

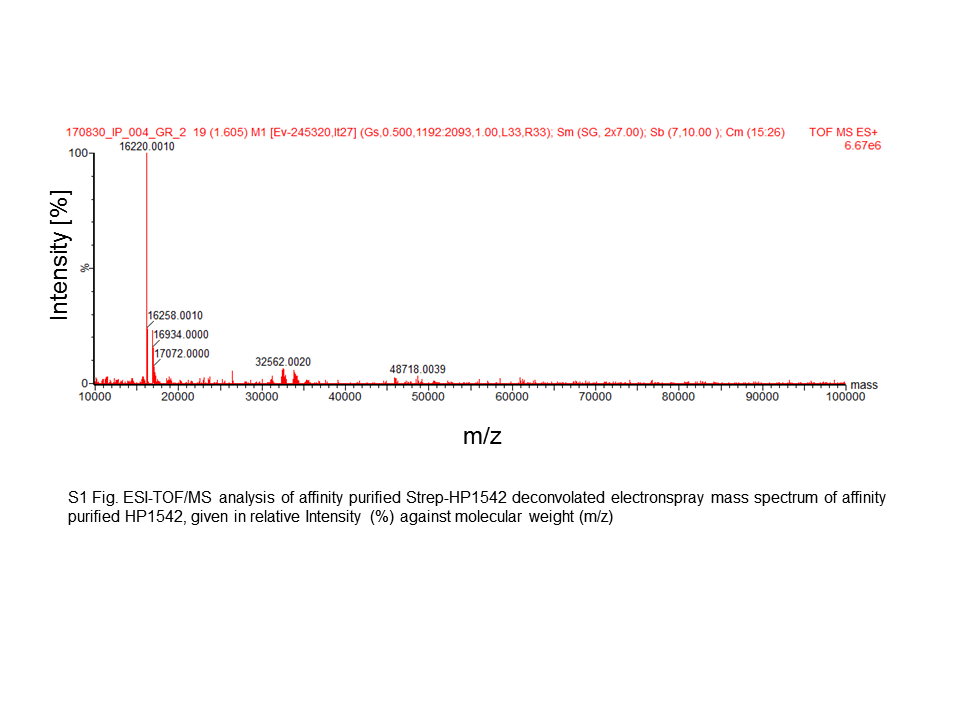

Supplement: S1 Fig — ESI-TOF/MS analysis of affinity purified Strep-HP1542 deconvolated electronspray mass spectrum of affinity purified HP1542, given in relative Intensity (%) against molecular weight (m/z). (TIF) [file pone.0218474.s001.tif]

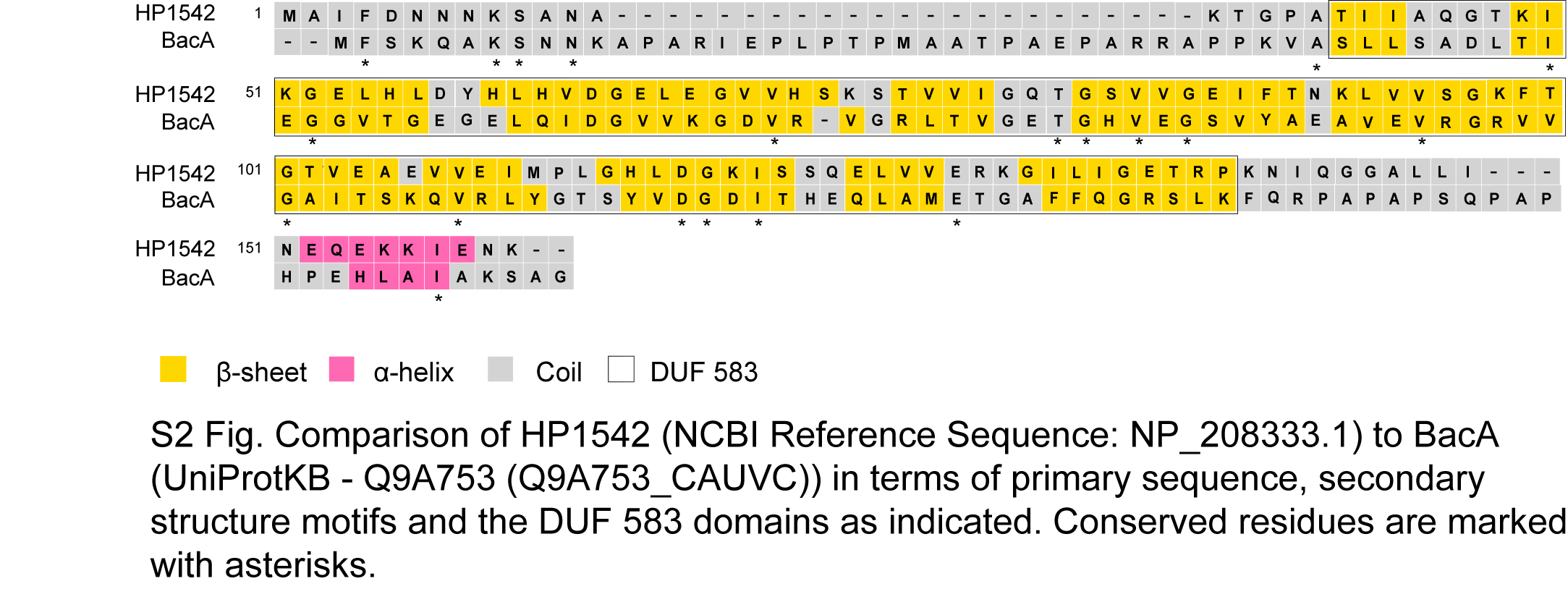

Supplement: S2 Fig — Comparison of HP1542 (NCBI Reference Sequence: NP_208333.1) to BacA (UniProtKB—Q9A753 (Q9A753_CAUVC)) in terms of primary sequence, secondary structure motifs and the DUF 583 domains as indicated. Conserved residues are marked with asterisks. (TIF) [file pone.0218474.s002.tif]

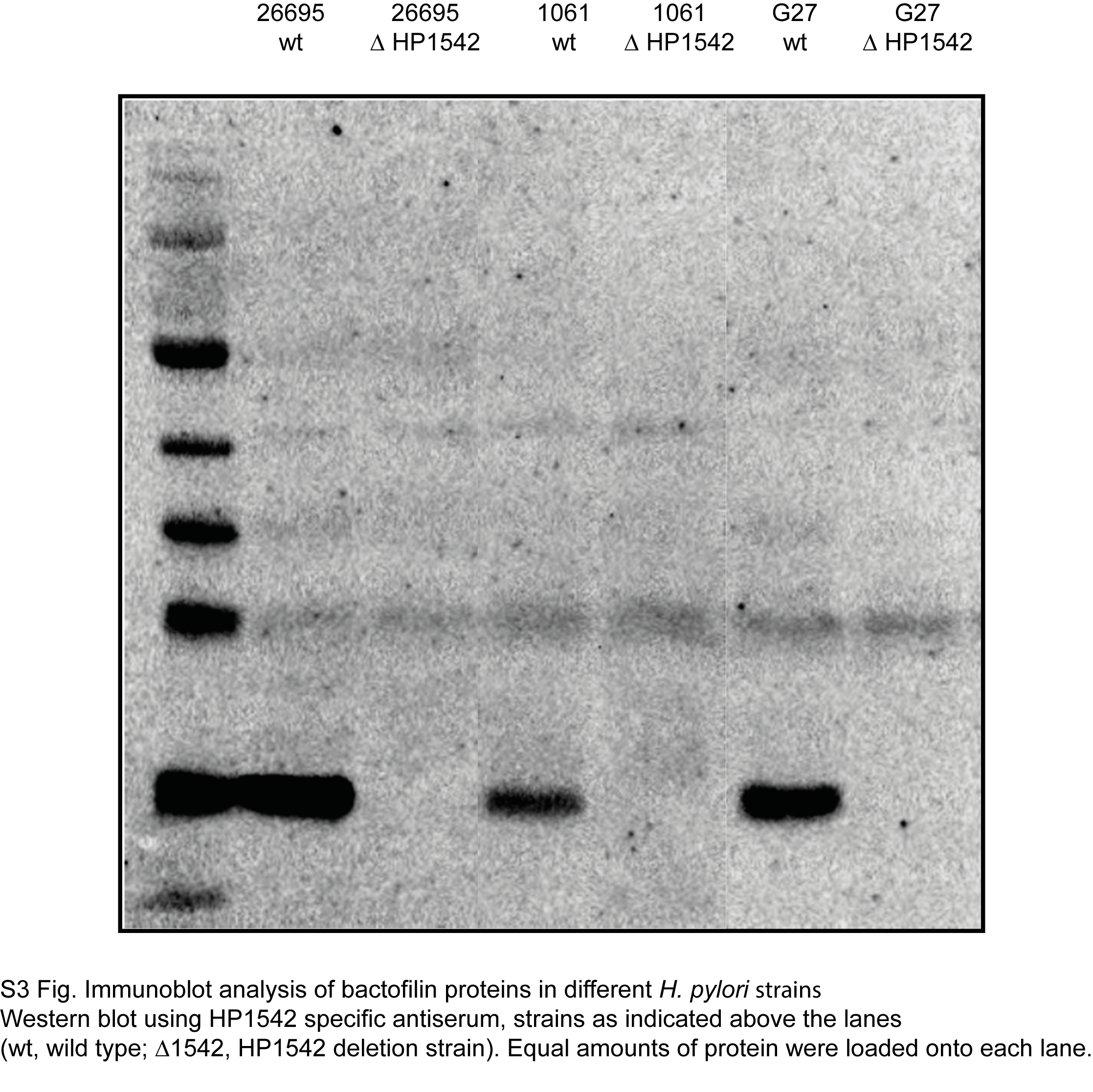

Supplement: S3 Fig — Western blot using HP1542 specific antiserum, strains as indicated above the lanes (wt, wild type; Δ1542, HP1542 deletion strain). Equal amounts of protein were loaded onto each lane. (TIF) [file pone.0218474.s003.tif]
